# New thinking for the next generation of antimalarials

**DOI:** 10.1038/s44321-026-00515-4

**Published:** 2026-09-02

**Authors:** Hannah Asiki, Jason Hlozek, Kathryn J Wicht, John G Woodland, Kelly Chibale

**Affiliations:** 1 Holistic Drug Discovery and Development (H3D) Centre, University of Cape Town, Rondebosch, Cape Town, 7701 South Africa; 2 Department of Chemistry, University of Cape Town, Rondebosch, Cape Town, 7701 South Africa; 3 South African Medical Research Council Drug Discovery and Development Research Unit, Department of Chemistry and Institute of Infectious Disease and Molecular Medicine, University of Cape Town, Observatory, 7925 South Africa

**Keywords:** Biotechnology & Synthetic Biology, Microbiology, Virology & Host Pathogen Interaction, Pharmacology & Drug Discovery

## Abstract

Despite decades of intensive research and substantial clinical gains, malaria remains a major global health challenge exacerbated by the continued emergence of drug-resistant strains of the causative agent, *Plasmodium* parasites. Encouragingly, recent advances in functional genomics, chemical biology and computational science are reshaping antimalarial drug discovery. In this review, we examine the discovery and development of next-generation antimalarials, including advances in phenotypic and target-based screening, omics-enabled target discovery and emerging therapeutic modalities such as long-acting agents, targeted covalent inhibitors and host-directed therapies. We further evaluate the opportunities and limitations of drug repurposing and discuss how artificial intelligence and data-driven approaches are reshaping target identification, compound optimisation and clinical development. Finally, we argue that future success will depend not only on scientific innovation but also on interdisciplinary collaboration, equitable partnerships, open science and the development of accessible therapies tailored to malaria-endemic populations.

## Abstract (web summary)

In this review, Kelly Chibale, John Woodland, Kathryn J. Wicht and colleagues discuss how functional genomics, chemical biology, artificial intelligence, and emerging therapeutic modalities are transforming antimalarial drug discovery and outline the scientific and collaborative strategies needed to deliver the next generation of accessible malaria therapies.

## Graphical abstract

**Figure:**
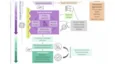

## Introduction: why do we still need new antimalarials?

Despite considerable progress over the past two decades, malaria remains a major global health burden and a persistent challenge for molecular medicine. The most recent estimates indicate that there were approximately 282 million malaria cases and 610,000 deaths worldwide in 2024, with the vast majority occurring in sub-Saharan Africa and disproportionately affecting children under 5 years of age (WHO, 2025). Although more than two billion cases and over 12 million deaths have been averted since 2000, progress has stalled in recent years, with case numbers increasing and incidence rising since 2015. This stagnation reflects a convergence of biological, environmental and systemic pressures, including climate change and population growth, as well as the concerning emergence and spread of antimalarial drug resistance.

Artemisinin-based combination therapies (ACTs), the cornerstone of current malaria treatment, are increasingly threatened by resistance. Alarmingly, artemisinin partial resistance has now been confirmed or suspected in multiple countries in Africa, accompanied by the declining efficacy of partner drugs (WHO, 2025). Historically, the spread of resistance to earlier antimalarials such as chloroquine and sulfadoxine-pyrimethamine (SP) led to dramatic increases in malaria mortality, underscoring the fragility of current therapeutic gains. The continued evolution of *Plasmodium* parasites, combined with widespread drug pressure, raises the realistic prospect that current frontline therapies may become ineffective within the foreseeable future.

**Figure 1:**
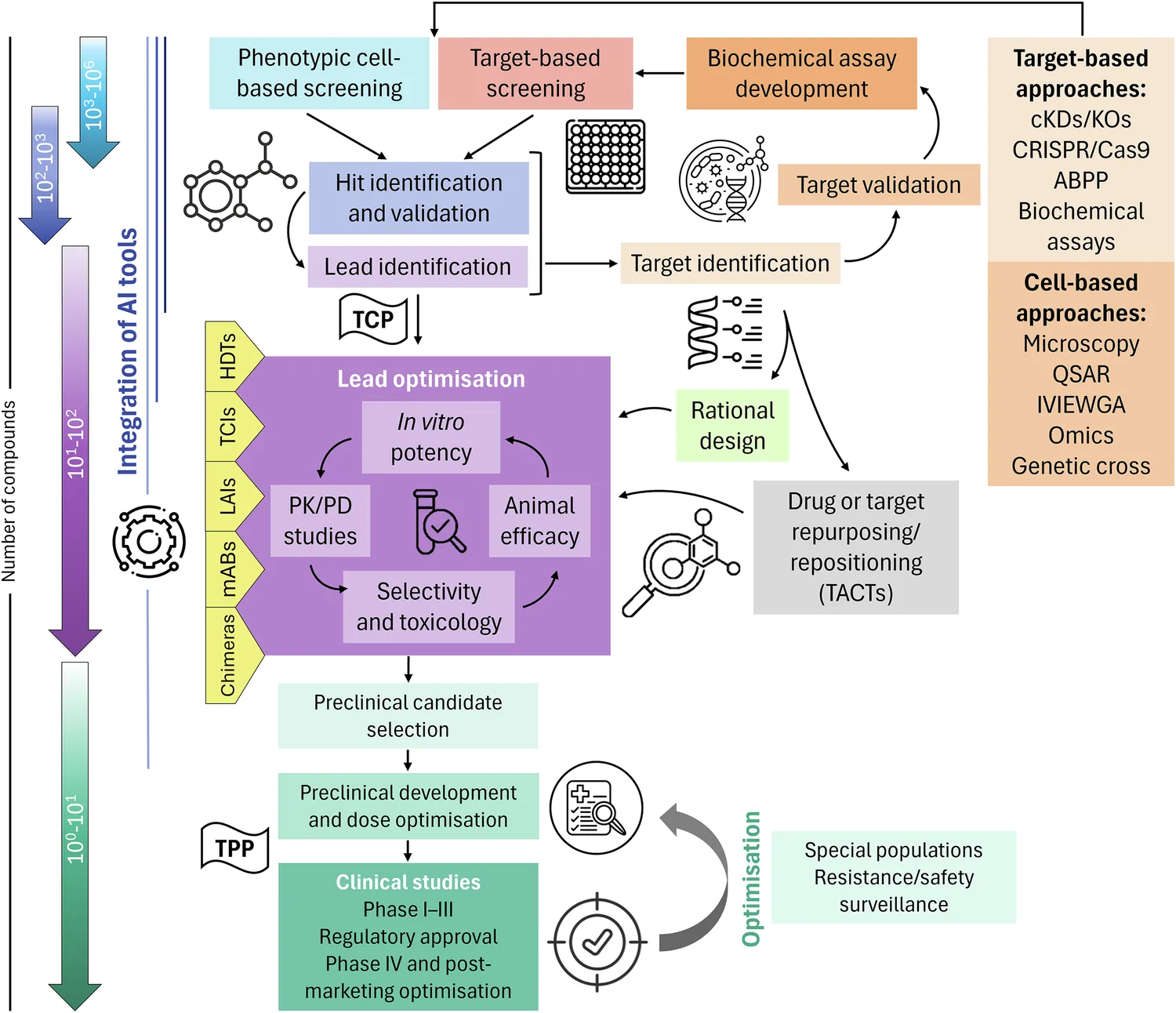
An illustration of the evolving antimalarial drug discovery and development landscape. Thousands to millions of compounds are typically employed in phenotypic or target-based high-throughput screening; these are eventually whittled down to dozens of compounds that undergo lead optimisation—with only a handful making it all the way through the clinic. Complementary strategies such as target validation, rational drug design, drug repurposing and biochemical assay development, as well as newer modalities such as host-directed therapies (HDTs), targeted covalent inhibitors (TCIs), long-acting injectables (LAIs) and monoclonal antibodies (mABs) will be critical to enable the flow of potential new medicines through this pipeline.

The contemporary antimalarial pipeline (Fig. 1) should be viewed against a remarkable transformation in malaria drug discovery over the past two to three decades. At the turn of the century, the pipeline of genuinely novel antimalarial medicines was sparse, reflecting limited commercial incentives and declining pharmaceutical-sector investment in diseases predominantly affecting low- and middle-income countries. The establishment of the Medicines for Malaria Venture (MMV) in 1999 and subsequent growth of public-private and philanthropic investment helped to change this landscape, creating a coordinated discovery and development network spanning academia, industry and endemic-country partners. This investment, together with advances in parasite culturing methods, high-throughput phenotypic screening and molecular technologies, contributed to what has been described as a renaissance in antimalarial drug development, generating new chemical classes and mechanisms of action (MoAs) alongside improved combinations of existing medicines (Anthony et al, 2012; Flannery et al, 2013; Hooft van Huijsduijnen et al, 2019).

However, a strong pipeline does not diminish the need for continued innovation. Antimalarial discovery and development remain lengthy and vulnerable to attrition (Wouters et al, 2020), while parasite resistance can progressively erode the therapeutic lifetime of otherwise highly effective medicines. Moreover, the ambition for new antimalarials has expanded considerably beyond replacing existing blood-stage treatments. Contemporary target candidate and product profiles reflect these broader ambitions, prioritising novel MoAs, high barriers to resistance, activity across multiple parasite life cycle stages, prevention of transmission and relapse, and simplified or long-acting regimens suitable for treatment and chemoprotection (Burrows et al, 2017). Together with the identification and validation of new molecular targets, the increasingly ambitious profiles are now driving the development of next-generation antimalarial strategies.

On the other hand, drug repurposing offers an additional complementary approach, leveraging existing pharmacological knowledge to accelerate development timelines and reduce costs. However, its success depends on overcoming challenges related to dosing, pharmacokinetics and target relevance. Finally, the integration of artificial intelligence (AI) and data-driven methodologies is beginning to reshape the antimalarial drug pipeline, enabling more efficient target identification, compound optimisation and clinical trial design. Together, these approaches point toward a more integrated and multidisciplinary paradigm for antimalarial drug discovery to outpace resistance and reinvigorate global malaria control efforts.

## An overview of the biological toolkit for developing next-generation antimalarials

### Life cycle complexity of *Plasmodium* and implications for drug discovery

*Plasmodium* parasites have a complex life cycle from the human host to the mosquito vector and back again. The five human-infecting *Plasmodium* species share similar life cycles, exemplified by *P. falciparum*.

Transmission begins when a female *Anopheles* mosquito injects sporozoites into the skin, which migrate via the blood to the liver. Crossing Kupffer cells, they invade hepatocytes and undergo asexual replication for about a week, forming schizonts that release ~20,000 merozoites into circulation (Frevert, 2004; Wirth and Pradel, 2012). These rapidly invade erythrocytes, initiating the 48-h blood cycle responsible for malaria symptoms (Crutcher and Hoffman, 1996). Parasites progress from ring forms to trophozoites and schizonts, fuelled by haemoglobin degradation and producing haemozoin crystals (malaria pigment) (Francis et al, 1997). Merozoites egress from schizonts, perpetuating parasitaemia (Wirth and Pradel, 2012). A fraction of merozoites undergo gametocytogenesis, producing mature crescent-shaped gametocytes after roughly 10 days (Bousema and Drakeley, 2011). These circulate in the blood for ~7 days where they can be ingested by a mosquito, mature into gametes, undergo fertilisation and form motile ookinetes (Angrisano et al, 2012). Penetrating the gut wall, ookinetes develop into oocysts that generate sporozoites, which migrate to salivary glands, completing the cycle (Ouologuem et al, 2023).

Given the extensive biological transformation of the parasite as it progresses through the stages, drug discovery for malaria is inherently multifaceted, wide-ranging and defined by the specific stages being targeted. Historically, most drugs have targeted the asexual blood stage (ABS) which causes clinical symptoms and is easiest to study in the laboratory (Swearingen and Lindner, 2018). However, with global efforts toward elimination, new drugs must overcome resistance, simplify dosing and provide longer-lasting protection. This shift has expanded the diversity of compounds in development, guided by target candidate and target product profiles (TCPs and TPPs) (Siqueira-Neto et al, 2023). For case management, combinations are sought that treat blood-stage infection (TCP-1), prevent relapse (TCP-3) and reduce transmission (TCP-5). For chemoprotection during outbreaks, TCP-4—activity against hepatic schizonts—is vital, and is often paired with TCP-1 to suppress new infections. Additionally, endectocides (TCP-6), which kill mosquitoes feeding on treated individuals, offer a novel transmission-blocking strategy. Together, these approaches highlight the need for therapies that address both disease treatment and malaria eradication.

### Traditional approaches to antimalarial drug discovery

Antimalarial drug discovery has traditionally relied on phenotypic screening, in which compounds are tested directly on whole parasites without prior knowledge of their molecular targets (Antonova-Koch et al, 2018; Guiguemde et al, 2010). This method has been highly effective because it measures functional outcomes in parasite culture and can reveal complex biology, including novel MoAs (Hovlid and Winzeler, 2016). Once a promising compound is identified, unravelling its precise MoA and pinpointing the drug target is often difficult, time-consuming and resource-intensive, although this knowledge is not required for regulatory approval (Swinney, 2013). Notably, ultra-high-throughput screening (HTS) campaigns against extensive compound libraries have previously been conducted with ABS parasites (Gamo et al, 2010), while the capacity to screen against other *Plasmodium* life cycle stages is orders of magnitude lower (Hovlid and Winzeler, 2016).

In contrast, target-based drug development begins by selecting a specific protein or pathway believed to be essential for parasite survival. Compounds are then screened or optimised to act against this target (Guerra and Winzeler, 2022). For a protein to be considered “druggable”, it must contain a binding pocket suitable for small molecules or antibodies, differ sufficiently from human proteins to ensure selectivity, and preferably be expressed across multiple parasite life cycle stages (Arendse et al, 2021). Ideally, it should also resist mutation under drug pressure to minimise the risk of resistance. This approach can also involve multi-target strategies in which inhibiting more than one protein produces synergistic effects or creates a “resistance trap”, making it harder for the parasite to adapt (Stokes et al, 2019a; Wicht et al, 2020).

Despite these theoretical advantages, translating target-based inhibitors into effective whole-cell activity has proven challenging. Progress has been slow, as optimising pharmacological properties and ensuring clinical relevance remains a complex and risky endeavour (Hovlid and Winzeler, 2016). This contrast between phenotypic and target-based approaches underscores both the promise and the difficulties of modern antimalarial drug discovery. Nevertheless, the identification of antimalarial drug targets has largely followed phenotypic screening, where compounds with strong activity against parasites are first discovered and their MoAs are then investigated afterwards (Godinez-Macias et al, 2025b).

An important classical approach used to retrospectively determine the target of existing antimalarials is a genetic cross, whereby male and female gametes fuse inside the mosquito gut, undergoing meiosis and gene mixing (Vendrely et al, 2020; Wellems et al, 1990). The resulting progeny are then isolated and analysed to map specific genes linked to survival, drug resistance and potential target pathways. However, the execution of genetic crosses in *P. falciparum* is technically challenging and resource-intensive; therefore, only four experimental crosses were completed during the first four decades of *P. falciparum* genetics research (Button-Simons et al, 2021; Vaughan et al, 2015). However, recent advances using humanised mouse models have enabled several additional crosses, facilitating resistance trait mapping for drugs such as piperaquine and quinine (Kanai et al, 2026; Kane et al, 2024).

In the context of current drug development, one of the most effective strategies for uncovering targets is in vitro evolution and whole-genome analysis (IVEWGA) (Luth et al, 2018). In this approach, parasites are cultured under drug pressure until resistant strains emerge. These resistant parasites are cloned, sequenced and compared to the original sensitive strain to identify mutations such as single-nucleotide variants or copy number variants (Ng and Fidock, 2019). Typically, these mutations occur in genes encoding the target of the drug, which can then be validated using tools such as CRISPR/Cas9 (Lee et al, 2019). This unbiased method is considered the gold standard for target discovery and has been central to the Malaria Drug Accelerator (MalDA) consortium, leading to the identification of several key targets (Forte et al, 2021a).

Examples include the *Plasmodium* P-type cation ATPase, *Pf*ATP4 (target of cipargamin or KAE609); dihydroorotate dehydrogenase, *Pf*DHODH (DSM265); translation elongation factor 2, eEF2 (DDD107498); acetyl-CoA synthetase, *Pf*ACS (MMV019721 and MMV084978) and phosphatidylinositol 4-kinase, *Pf*PI4K (MMV390048)—all essential for parasite survival across different stages (Luth et al, 2018). IVEWGA has also revealed *Plasmodium* aminoacyl-tRNA synthetases (aaRSs) as critical targets (Xie et al, 2023). These enzymes drive protein synthesis in multiple parasite organelles. Inhibitors such as halofuginone (prolyl-tRNA synthetase), cladosporin (lysyl-tRNA synthetase) and benzoxaboroles (leucyl-tRNA synthetase) show potent activity, although none has advanced to clinical trials due to challenges with stability, bioavailability and selectivity (Khan, 2016).

Despite its success, IVEWGA can be time-intensive, and some compounds are “irresistible”, meaning that they fail to generate resistant strains. Such compounds may act on multiple targets or non-mutable pathways, making them highly valuable as they are less prone to resistance. To study these, researchers employ complementary methods, including biochemical assays (e.g., haemozoin formation inhibition), fluorescence microscopy and testing against conditional knockdown lines (Langeveld et al, 2025). Additionally, omics-based approaches such as proteomics and transcriptomics have expanded target discovery (Cowell and Winzeler, 2019; Mohammed et al, 2024; Painter et al, 2018); these approaches will be discussed later in this review.

A variety of chemoproteomics methods have been useful in determining drug targets (Challis et al, 2022; Lu et al, 2021b). For instance, covalent immobilisation and pull-down experiments helped validate *Pf*PI4K as the target of MMV390048 (Paquet et al, 2017b) and to identify purvalanol B as a casein kinase 1 binder (Knockaert et al, 2000). Activity-based protein profiling (ABPP) was employed to identify the essential α/β serine hydrolase monoacylglycerol lipase (*Pf*MAGLLP) as the target of a salinipostin A (Yoo et al, 2020). Furthermore, a cellular thermal shift assay coupled with mass spectrometry (MS-CETSA) studies revealed that quinine, one of the oldest antimalarials, binds to the purine nucleoside phosphorylase (*Pf*PNP) (Dziekan et al, 2019).

Biochemical assays have also been useful in supporting target identification studies. For example, the *Pf*Ark1 (*a*urora-related kinase 1) inhibitors hesperidin (Langeveld et al, 2025) and CHIR-124 (Wicht et al, 2025), the cGMP-dependent protein kinase (*Pf*PKG) inhibitor ML10 (Baker et al, 2017) and the *Pf*PI4K inhibitors AZD0156 (Woodland et al, 2025), MMV390048 (Paquet et al, 2017b) and KDU691 (McNamara et al, 2013) were all shown to bind to recombinantly expressed versions of these enzymes. Together, these strategies highlight the evolving toolkit for antimalarial drug discovery. While phenotypic screening remains the foundation, integrating genomic, biochemical and proteomic methods has broadened the landscape of validated targets, offering new opportunities to combat resistance and discover new therapeutic strategies.

However, the criteria for next-generation therapies are stringent. Since no fully protective vaccine exists, chemoprevention remains essential for vulnerable groups, including travellers, pregnant women and children under the age of five (Greenwood et al, 2021). Long-acting oral and injectable drugs are expected to form the backbone of chemoprevention, addressing current barriers such as frequent dosing, limited options in early pregnancy and challenges for women living with HIV. Ideally, these drugs should also target multiple parasite life cycle stages (Braunack-Mayer et al, 2026; El Gaaloul et al, 2024). Drug development must also consider tropical climate conditions, ensuring chemical stability in heat and humidity. Because children are the primary population affected, formulations must be child-friendly, with taste-masking and dispersibility, alongside rigorous pharmacovigilance to account for genetic variations in drug metabolism (Siqueira-Neto et al, 2023). Seasonal malaria chemoprevention (SMC) is particularly critical, yet resistance to sulfadoxine-pyrimethamine-amodiaquine necessitates new alternatives. Effective SMC drugs should combine high potency, safety, long half-lives and complementary MoAs to enable monthly dosing (Plowe, 2022).

Unlike diseases predominantly afflicting wealthier nations, antimalarial drug development faces unique challenges: high costs, limited commercial returns and reliance on public procurement through national malaria control programmes and agencies such as the Global Fund (Hanefeld, 2014). These realities underscore the need for sustained innovation and global collaboration to deliver safe, effective and accessible therapies.

## Can drug repurposing help to deliver new antimalarials?

Drug repurposing is a strategy in which existing medicines are used to treat diseases for which they were not originally designed, sometimes involving further structural modification of the compound (drug repositioning). One of the biggest benefits of this approach is a significant reduction in the drug development timeframe and an associated reduction in cost, as the safety and tolerability profile of the active component is often already well established. Repurposing and repositioning are enticing approaches for diseases such as malaria, in which drug development is not commercially attractive. The potential for faster regulatory approval and implementation, therefore, offers an alternative path to traditional de novo drug discovery.

### Approaches to drug repurposing

#### Phenotypic screening

Identifying suitable drugs for repurposing is a key step in the success of this approach, which has often employed HTS of compound libraries for activity against *Plasmodium*. As described above, traditional screening has focused on phenotypic responses, as this provides an unbiased way to identify compounds with varied targets acting on different life cycle stages of the parasite. Most phenotypic-response assays have been developed against the ABS, which was the basis for the MMV “boxes”—compounds prioritised by MMV for the development of new antimalarials (Adam et al, 2024; Spangenberg et al, 2013).

However, it is important not to overlook other parasite life cycle stages in HTS campaigns seeking new chemical matter to reposition. Gametocytes are an important bottleneck for transmission to the mosquito vector, and blocking this has been identified as a key target area (Burrows et al, 2017). New screens are continually being developed (D’Alessandro et al, 2016; Delves et al, 2018; Lucantoni et al, 2016; Miguel-Blanco et al, 2015; Paonessa et al, 2022; Plouffe David et al, 2016), including incorporating a mouse model for in vivo monitoring of transmission blocking (Brancucci et al, 2025). A recently developed transgenic Ookluc line has enabled HTS for compounds that inhibit parasite fertilisation, the initial step within the mosquito vector, which provides a new tool for compound identification (Calit et al, 2023).

Key chemical matter has also been identified from screening for in vivo activity against *Plasmodium* within the mosquito (Probst et al, 2025), which contributes to malaria control. Screening against liver stages has often focused on the liver schizonts and early hypnozoites (Maher et al, 2021a; Maher et al, 2021b; Nguyen et al, 2024; Roth et al, 2018), but recently the first HTS of dormant liver-stage hypnozoites has been established (Maher et al, 2025), which has been missing from the routine drug discovery pipeline and contributes towards finding a radical cure, i.e. complete removal of dormant parasites alongside ABS parasites to prevent disease relapse.

#### Drug target repurposing

In addition to repurposing existing drug compounds, drug targets themselves can also be repurposed. This target repurposing approach has been especially evident with kinase targets originally identified in cancer research, which are now being investigated in *Plasmodium* (Younis et al, 2012). The *Pf*PI4K inhibitor MMV390048 progressed to Phase IIa clinical trials (Mohammed et al, 2023; Paquet et al, 2017a), although the development of this compound was discontinued due to embryofoetal toxicity in rats (Demarta-Gatsi et al, 2022). In another target repurposing example, the anticancer compound sapanisertib (MLN0128), originally developed to target the human mTOR kinase, has demonstrated excellent activity across multiple parasite stages as a dual *Pf*PI4K and *Pf*PKG inhibitor (Arendse et al, 2022), with drug repositioning also being pursued (Gachuhi et al, 2025). Another success story is imatinib, an FDA-approved tyrosine kinase inhibitor that was recently discovered to block the egress of *Plasmodium* from erythrocytes (Kesely et al, 2016) and has been used in a clinical trial to enhance antimalarial efficacy when combined with artemisinin (Chien et al, 2021).

#### Target-based screening

A complementary strategy for drug repurposing is target-based screening, mentioned briefly above, which seeks to identify compounds active against validated *Plasmodium* targets, with subsequent confirmation through phenotypic validation. A list of priority targets has been released by MalDA, based on chemical and genetic validation, propensity to generate resistance, druggability, assay availability and structural information (Forte et al, 2021b). Although there is still a need to develop high-throughput screens specific to many of these targets, a few examples do exist. Screening for inhibitors of the mitochondrial enzyme *Plasmodium* dihydroorotate dehydrogenase (DHODH) identified compound DSM265 after structural optimisation (Baldwin et al, 2005; Margaret and Pradipsinh, 2010; Phillips et al, 2015), although this target has since been deprioritised as resistance has been found to arise relatively easily (Forte et al, 2021b). For kinase targets, a PKG HTS in combination with Kinobead studies has led to the discovery of a promising multi-stage targeting scaffold (Penzo et al, 2019), while five *Plasmodium* kinases were screened against a large library which demonstrated the capacity to selectively target *Plasmodium* kinases over mammalian ones (Crowther et al, 2016).

Combining multiple screening strategies can be a powerful way to more rapidly prioritise candidates for repurposing, particularly by leveraging computational methods to dock and screen compounds in silico against known targets or pathways. Target-specific computational screens have been developed for compounds targeting chemically-validated kinases (Godara et al, 2024), the haemozoin formation pathway via β-haematin formation (de Sousa et al, 2020), and DHODH (Pavadai et al, 2016), among others.

At this juncture, it is noteworthy that the potential for a drug to be repurposed requires demonstrating that a predicted efficacious dose for the new indication is within the already-established clinical safety and tolerability profile of the drug to be repurposed. This was recently demonstrated for malaria using a human dose prediction approach based on integrating PK-PD modelling with physiologically-based pharmacokinetics modelling to investigate the potential to repurpose an anticancer human kinase inhibitor (Redhi et al, 2026).

### Challenges of drug repurposing

Although drug repurposing is an appealing route to discovering new antimalarials, challenges remain. Despite open-access initiatives led by organisations such as MMV and collaborations across public and private sectors sharing compound identities, intellectual property restrictions are still in place for certain structures, with examples where companies patent whole compound series even if they are later discarded (Chesbrough and Chen, 2013). This disincentivises exploration of known chemical entities and was reported as the second most common barrier to repurposing after financial constraints (Krishnamurthy et al, 2022). Additionally, there can be restrictions to data sharing, meaning that abandoned projects and their corresponding results remain unpublished, restricting knowledge-sharing (Rogawski and Federoff, 2011). Another challenge is that the regulatory pipeline for repurposed drugs is less well defined than for novel compounds, introducing additional financial risk (Ivanov et al, 2024; Recino et al, 2025). Even after a suitable drug has been identified for repurposing, it must still undergo randomised clinical trials for malaria which can be logistically challenging, costly and time-consuming, particularly in the resource-limited settings with the highest burden of malaria (Espinoza, 2025). Although these events conspire to limit drug repurposing and repositioning efforts, a few successful examples, such as the kinase inhibitors discussed above, highlight the potential of this approach to uncover new antimalarial medicines.

## New frontiers and other promising approaches for next-generation antimalarials

The current landscape of drug discovery is increasingly shaped by modern omics-driven approaches, and malaria research is no exception. These cutting-edge technologies enable probing of fundamental *Plasmodium* biology to identify new drug targets. Recently, 540 novel, druggable targets were prioritised following inspection of the full *P. falciparum* genome to identify proteins essential for the ABS parasites with evidence of small molecule binding (Godinez-Macias et al, 2025a). The annotation used in this process is freely available at https://pftargetbrowser.org/ and downstream scoring by MalDA experts has put forward 29 potential new targets, with five particularly highlighted: aspartate transcarbamoylase (ATCase), topoisomerase I (TopoI), DNA gyrase subunit B (GyrB), glucose-6-phosphate dehydrogenase-6-phosphogluconolactonase (GluPho) and bromodomain protein 1 (BDP1). These can serve as a starting point for target-based drug discovery and it is likely that these will prove to be invaluable.

### Omics approaches to accelerate antimalarial drug discovery

#### Genomics

Omics approaches (genomics, transcriptomics, proteomics and metabolomics) have utility in elucidating the underlying MoA of active compounds, an important aspect of drug characterisation, and have been extensively reviewed (Carolino and Winzeler, 2020; Lu et al, 2021a; Okombo et al, 2021; Yu et al, 2021). A recent in-depth study combined genetic information from 724 *P. falciparum* resistance cell lines, uncovering novel resistance genes and enabling the identification of multi-drug resistance markers (Luth et al, 2024). This dataset contributes a starting point for prioritising irresistible chemical matter and unravelling the key mutations in genomics experiments for MoA deconvolution.

Genomic overexpression techniques involve the use of cell libraries engineered to systematically overexpress potential targets, in which overexpression can confer resistance under drug pressure if the target is directly engaged by the compound. Such libraries have been successfully applied to MoA deconvolution in other parasitic diseases such as leishmaniasis and trypanosomiasis (Begolo et al, 2014; Gazanion et al, 2016). Until recently, this has not been possible for malaria owing to the difficulty in large-scale genetic manipulation of *Plasmodium*, but the advancement in *P. knowlesi* transfection efficiency coupled with barcode sequencing and artificial chromosomes has enabled a cell overexpression library to be developed and validated with a PI4K inhibitor (Bower-Lepts et al, 2024). This tool is a novel addition to the target discovery pipeline, and it is likely to expedite the identification of MoAs of phenotypically discovered compounds in the future.

#### Transcriptomics and proteomics

The *Plasmodium* transcriptome can also contain valuable information about expression levels following drug perturbation, and this has been recently investigated with known antimalarial compounds which revealed distinct clustering dependent on compound MoA (Ishii et al, 2025). In one example, the *Plasmodium* transcriptome was screened to identify essential, multi-stage genes and their associated targets, after which appropriate chemical matter was identified; this helps to prioritise compounds for drug repurposing (Borba et al, 2023). Single-cell transcriptomics can similarly be applied to identify crucial proteins and predict likely drug structures to engage these (Choudhuri and Ghosh, 2024). As briefly mentioned above, a *Plasmodium-*specific cellular thermal shift assay (CETSA) has been developed to show definitive target engagement by detecting shifts in protein melting temperature upon target engagement, enabling proteome-wide profiling (Dziekan et al, 2020) and which has recently been improved (Pazicky et al, 2026).

#### Compound resistance generation

As resistance remains a concern for antimalarial drug development, it is important to consider the likelihood of resistance emerging for new scaffolds. This is well established by checking for minimum inoculum of resistance for promising new compounds, in the hope that in vitro resistance is not easily generated, although this does not always translate well to the field (Duffey et al, 2021). It is possible also to interrogate the target, demonstrated by the proof-of-concept study ResMAP, which mutated every amino acid around the binding pocket of lysyl-tRNA synthetase (KRS) to investigate resistance likelihood (Wall et al, 2024). One can envision applying this to other targets to identify key amino acid mutations conferring resistance, which in turn can guide compound design to circumvent these mechanisms.

### Morphology-based techniques for mode of action elucidation

Morphology-first approaches, in which changes in cell morphology are correlated to MoAs, are an upcoming trend in drug discovery and can also help predict toxicity. In mammalian cells, a cell painting approach was able to identify morphological changes upon drug perturbation across large compound screens, employing machine learning analysis (Wolff et al, 2025). A similar project is currently being undertaken in *Plasmodium* through a partnership between MMV, the University of Dundee and LPIXEL (Venture, 2025). Powerful combinations of cell painting with existing transcriptomics data can enable predictions of novel compounds in the absence of additional costly new transcriptomics data (Ha et al, 2025); it remains to be seen if this can be applied to *Plasmodium*.

### New modalities in drug discovery

#### Host-directed therapies

While traditional drug discovery has primarily focused on small molecules acting on *Plasmodium* protein targets, there are new modalities emerging that merit discussion. One such approach is host-directed therapy (HDT), in which compounds act on host rather than parasite targets. This can be especially valuable when pathways essential for *Plasmodium* development, such as invasion of erythrocytes and/or hepatocytes, are disrupted. Its application is confounded by the fact that different *Plasmodium* species use distinct receptors for host cell invasion (Manzoni et al, 2017) and there are marked differences in host susceptibility to invasion (Ebel et al, 2021), which perhaps accounts for the fact that this approach is yet to yield candidates for clinical development. Modulation of the immune response is an alternative HDT. In this context, the anti-inflammatory drug ruxolitinib has shown limited success when repurposed for acute malaria infection to suppress the immune response, potentially boosting immunity in future infections (Webster et al, 2025).

#### Targeted covalent inhibitors

A second new modality for antimalarials is targeted covalent inhibitors (TCIs); these are compounds in which the active component attaches covalently to an amino acid residue in the drug target, in contrast to traditional drugs that typically rely on weak, noncovalent binding kinetics to drive target engagement. This approach has potential advantages of better selectivity, longer duration of action and therefore lower resistance risk and lower required dosing, among others (Singh et al, 2011). Nine TCI drugs have been FDA-approved in the last decade for cancer treatment (Dhillon, 2023; Singh, 2022), and although no TCIs are in clinical trials yet for malaria, discovery efforts are ongoing. The proteasome has historically been a challenging target to drug, but was successfully targeted with TCIs, which demonstrated the expected benefit of decreased propensity for resistance generation (Stokes et al, 2019b). Brettell et al. further achieved target engagement with TCIs designed against cyclin-dependent-like protein kinase-3 (CLK3), providing the first proof-of-concept for covalent kinase inhibition in *Plasmodium* (Brettell et al, 2025; Brettell et al, 2024). In addition, CRISPR-based technologies can be applied to finding targetable amino acid residues, potentially driving interest in the TCI modality (Benns et al, 2022).

#### Long-acting injectables

In a push towards longer-lasting protection, long-acting injectables (LAIs) are an alternative modality. These are desirable because they can provide sustained prophylaxis by effectively behaving as a chemical vaccine. LAIs may also help reduce malaria transmission (Macintyre et al, 2018). Several compounds have been developed as LAIs, including MMV371, a prodrug of atovaquone already shown to provide 3 months of protection that is entering Phase Ib clinical trials (ClinicalTrials.gov; Gupta et al, 2024), and ELQ-331, a prodrug of ELQ-300/MMV055 (Karunakaran et al, 2024; Smilkstein et al, 2019).

#### Monoclonal antibodies

Monoclonal antibodies (mABs) are another modality for long-lasting protection (Aleshnick et al, 2022). Advantages of mABs include their high specificity, which makes them amenable to combination therapy, and likely favourable safety profiles over traditional small molecules. However, one current limitation of mABs is their high cost. For malaria, a key consideration is the complex life cycle of *Plasmodium* in comparison to the viral diseases for which mABs have been highly successful. An ideal candidate would act on the pre-erythrocytic stages, as achieving protection at this point is generally more feasible than attaining sterile clearance of blood stage parasites. One example is CIS43LS, a monoclonal antibody in Phase II clinical trials that targets the circumsporozoite protein (CSP), key for sporozoite invasion of hepatocytes (Skinner et al, 2025). CSP has attracted significant attention because the truncated form is a component of the newly approved RTS,S/AS01 and R21/Matrix-M vaccines (Singer et al, 2024). Another sporozoite protein of interest is thrombospondin-related adhesive protein (TRAP), although using TRAP combined with the RTS,S vaccine did not show an additional benefit (Kester et al, 2014). In the erythrocytic stages, reticulocyte-binding protein homologue 5 (RH5) has emerged as the leading target due to its essentiality to merozoite invasion of erythrocytes, with anti-*Pf*RH5 showing promise in monkeys (Douglas et al, 2019).

#### Chimeric molecules

Finally, among the most promising innovations are chimeric molecules, particularly proteolysis targeting chimaeras (PROTACs), which harness cellular proteolytic machinery to degrade essential proteins (Birkholtz et al, 2026). Unlike traditional small molecules, these agents can act on previously “undruggable” targets, reduce required therapeutic concentrations and slow resistance development (Ng et al, 2017).

#### Precision medicine

The highest malaria burden is in sub-Saharan Africa, which has the most genetically diverse populations yet remains comparatively under-characterised. This presents a particular challenge for precision and/or stratified medicine in which patient genetics, environmental circumstances and lifestyle should be factored into treatment decisions. Limited understanding, along with high costs and practical constraints, still remain to be overcome before precision and/or stratified medicine can become a reality for malaria (Okeke and Ksouri, 2026).

### Combination therapies

Combination therapies form the cornerstone of the current frontline drugs and, as already mentioned earlier, usually involve combining fast-acting artemisinin with a slower-acting partner drug to form ACTs (WHO, 2023). However, the emergence of partial resistance to ACT regimens has prompted consideration of adding a third drug, forming triple ACTs (TACTs) as a strategy to delay resistance development. These regimens are already being evaluated in trials across Asia and Africa (van der Pluijm et al, 2020). Mathematical modelling has been applied to assess the benefits of TACTs, as the long-term evolutionary dynamics of resistance cannot be assessed with clinical trials. These indicate that early implementation of TACTs, especially in Africa, where artemisinin resistance is relatively low, could have a marked reduction in treatment failure rates (Nguyen et al, 2023).

### Clinical development and dose optimisation

The development of a next-generation antimalarial does not end with the identification of an efficacious molecule or even regulatory approval. Selection of an appropriate dose and formulation across the populations in which the drug will ultimately be deployed is equally important, particularly because malaria disproportionately affects young children and pregnant women, in whom PK can differ substantially from that of non-pregnant adults. Historically, PK data in these populations have often been limited at the time dosing recommendations were established. For example, subsequent population PK analysis of sulfadoxine-pyrimethamine (Fansidar) showed that children aged 2–5 years had approximately half the drug exposure observed in adults after adjustment for dose, suggesting that contemporary paediatric dosing recommendations were suboptimal (Barnes et al, 2006). For tafenoquine, modelling of pre-registration trial data indicated a clear dose-response relationship and suggested that the licensed 300 mg adult dose achieves substantially less than the maximal obtainable hypnozoiticidal effect, although the safety and efficacy of higher doses require prospective evaluation (Watson et al, 2022). Thus, clinical pharmacology should be considered a continuum extending from early development through Phase I–III trials to post-marketing surveillance and dose optimisation (Fig. 1). For next-generation antimalarials, early inclusion of children, pregnant women and other priority populations in appropriately designed PK/PD studies will be critical to ensure that innovative medicines translate into optimal real-world efficacy.

## AI and data-driven innovation in antimalarial drug discovery

Artificial intelligence (AI) is increasingly being applied within the drug discovery pipeline, enabling the integration of heterogeneous datasets to enable analysis and prospective predictions which may not be readily apparent (Ferreira and Carneiro, 2025). Indeed, the number of clinical candidates that have employed AI during their development continues to grow (Jayatunga et al, 2024). Frequent areas of application include improving target identification, compound optimisation and clinical development. Here, we highlight several common areas of application with relevant examples.

### AI-enabled target identification and validation

As described above, a central challenge at the start of the drug discovery process is the identification of novel drug targets within complex biological systems which can unlock new treatment options. Multi-modal deep learning models, such as the PandaOmics platform (Kamya et al, 2024), can integrate knowledge from multi-omics datasets and gene interaction networks to prioritise genes associated with disease (Pun et al, 2023). In addition, large-language models (LLMs), such as Google’s Co-Scientist and OpenAI’s Deep Research (Moons et al, 2025), can efficiently mine large bodies of scientific literature and identify unrecognised connections to support novel target identification and assist with target deconvolution for compounds with unknown MoAs. Computer vision models provide an alternative approach for target deconvolution by comparing cellular phenotypes induced by novel compounds to reference profiles of compounds with known MoAs (Chandrasekaran et al, 2021). Following target identification, tools such as AlphaFold can predict the structure of identified protein targets and facilitate downstream identification of druggable pockets for which generative AI methods can then propose chemical starting points by optimising chemical structures against predicted binding interactions (Adams et al, 2025).

### AI-aided compound discovery and optimisation

Quantitative structure-activity relationship (QSAR) models learn relationships between chemical structure and various biochemical parameters to efficiently guide multi-parameter compound optimisation (Turon et al, 2023; Zhang et al, 2025). Complementary approaches assess synthetic feasibility and propose optimised synthetic routes (Tu et al, 2025) while ligand-based generative models, such as the REINVENT package (Loeffler et al, 2024), can explore novel chemical space. Though AI tools typically require technical expertise to use, platforms such as the Ersilia Model Hub (Turon, 2024) and Drug Discovery 4 Global Health (DD4GH) (Nasto, 2025) are lowering barriers to adoption by providing accessible, no-code implementations of these tools.

### AI in clinical design and implementation

Clinical trials represent the final and most costly stage of the drug development pipeline. While the application of AI for an antimalarial clinical trial has not yet been reported, AI methods are increasingly being applied in other therapeutic areas such as oncology (Askin et al, 2023). As AI approaches are often transferable between disease areas, examples from other disease areas can provide a foundation for potentially improving the design of future antimalarial clinical trials. The most frequent application is to support patient recruitment (Askin et al, 2023) by integrating digital clinical records with unstructured medical data to identify eligible patients more efficiently (Beck et al, 2020). AI is also leveraged to optimise trial design, including cohort composition and endpoint selection to improve prediction success (Aliper et al, 2023) and to maximise the statistical power of trial findings. Additional applications include data analysis of trial results and real-time monitoring of participants to predict patient dropout to enable early intervention.

As indicated above, a significant challenge in conducting clinical trials for antimalarial treatment is the high genetic diversity of populations in endemic regions. Variation in pharmacogenes can lead to substantial differences in drug response (Haga, 2024; Zack et al, 2025). This remains acutely understudied in Africa, where over 60% of malaria clinical trials occur in populations possessing the greatest genetic diversity of any continent (Gomez et al, 2014; Takata et al, 2020). To address this, a collaboration between the H3D Centre at the University of Cape Town and the Ersilia Open Source Initiative analysed data from the PharmGKB database to identify variably expressed pharmacogenes in African populations and identified drug-gene pairings for common drugs used to treat malaria and tuberculosis. In that work, machine learning (ML) and AI were coupled to pharmacometrics modelling to hypothesise pharmacogenes of potential clinical interest, with the goal to propose dose adjustments for better treatment outcomes (Turon et al, 2025).

### Challenges for AI integration into antimalarial drug discovery

Despite these advances, applications of AI in antimalarial drug discovery remain more limited than in other disease areas (Leung et al, 2026; Odah, 2025). Key challenges include data scarcity, complex parasite biology, limited research resources and the need for context-specific deployment. Insufficient high-quality training data, a common limitation in resource-constrained settings, reduces model performance and generalisability beyond the training data. Meanwhile, mechanisms to connect data silos and provide incentives for sharing data have been explored in the Global North. For example, the malaria inhibitor prediction (MAIP) platform was developed through standardised model training across partners with subsequent model sharing for mutual benefit from a broader chemical space without exposing proprietary data (Bosc et al, 2021). Alternatively, federated learning approaches enable securely pooling data through a trusted third-party platform to facilitate model training with a broader domain of applicability while preserving data privacy and benefit sharing across contributors (Napoli et al, 2025).

Overall, AI and data-driven approaches provide a powerful framework for accelerating antimalarial drug discovery. However, their impact will depend on investment in data generation, improved data sharing infrastructure and careful integration into experimental workflows to translate into real-world benefit for the costly research and development process. Mutually-beneficial data-sharing methods should be encouraged in antimalarial research to unlock the field’s full potential and bridge the divides between established and emerging research centres.

## Conclusion: What will a successful next-generation antimalarial look like?

The future of antimalarial drug development will depend not only on discovering new compounds but also on rethinking how antimalarial therapies are identified, optimised and deployed. As discussed throughout this review, the next generation of antimalarials must move beyond the historical focus on rapid clearance of ABS parasites toward integrated, multi-stage interventions capable of supporting malaria elimination. A successful next-generation antimalarial will therefore likely combine several key characteristics: activity across multiple parasite life cycle stages, a high barrier to resistance, compatibility with long-acting or simplified dosing strategies, and affordability and relevance for widespread implementation in endemic regions.

Ultimately, effective malaria medicines require fixed-dose drug combinations to prevent resistance and enhance compliance. However, discovery begins with single compounds before identifying optimal partners. Advances in parasite biology and target discovery are already reshaping the field. The expansion of genomic, transcriptomic, proteomic and chemoproteomic toolkits has dramatically increased the number of validated and potentially druggable targets in *Plasmodium* parasites. Approaches such as IVEWGA, CRISPR-based validation, CETSA profiling, overexpression libraries and morphology-based phenotypic screening are accelerating MoA deconvolution and helping to identify compounds with reduced resistance liability. Importantly, these approaches are broadening the therapeutic landscape beyond conventional small molecules, enabling the exploration of emerging modalities including TCIs, HDTs, PROTACs, mABs and LAI chemoprotection. Collectively, these developments signal a transition from empirical discovery toward increasingly mechanism-informed and systems-level antimalarial development.

At the same time, the review highlights that no single discovery strategy is likely to be sufficient in isolation. Phenotypic screening remains a cornerstone of antimalarial discovery because of its ability to identify biologically active compounds in unbiased cellular contexts, yet target-based approaches provide critical mechanistic insight and opportunities for rational optimisation. Increasingly, the most effective paradigm is likely to emerge from synergy between experimental and computational approaches. AI and machine learning are beginning to augment nearly every stage of the discovery pipeline; however, AI is not a substitute for experimental biology. Rather, its value lies in integrating complex datasets, identifying hidden relationships and guiding iterative cycles of empirical validation. In malaria research specifically, the impact of AI will depend heavily on the generation of high-quality datasets, context-specific modelling and closer integration with wet-lab workflows.

Drug repurposing similarly represents a complementary rather than a standalone solution. As discussed above, repurposed compounds and targets—including kinase inhibitors originally developed for oncology—have demonstrated the potential to accelerate discovery timelines and uncover novel mechanisms of action. Yet repurposing also illustrates many of the broader challenges facing antimalarial development, including pharmacokinetic incompatibility, toxicity concerns, intellectual property barriers and uncertain regulatory pathways. Consequently, the future pipeline will likely depend on balancing repurposing strategies with continued investment in genuinely novel chemical matter and first-in-class mechanisms.

Finally, scientific innovation alone will not determine success. Malaria disproportionately affects populations in low- and middle-income countries where financial return on drug development remains limited. As a result, sustained progress will require strong public–private partnerships, open-science initiatives and globally coordinated funding mechanisms. Organisations such as MMV and collaborative consortia such as MalDA demonstrate the power of precompetitive collaboration, shared compound libraries and collective target prioritisation. Expanding equitable access to data, infrastructure and leadership opportunities—particularly for researchers and institutions in endemic regions—will be essential both ethically and scientifically. The increasing use of AI further reinforces the importance of equitable data sharing and representation, particularly given the underrepresentation of African populations in pharmacogenomic and clinical datasets.

Ultimately, a successful next-generation antimalarial is unlikely to be defined by a single “breakthrough” molecule. Rather, it will emerge from an interdisciplinary and globally- connected innovation ecosystem that integrates molecular parasitology, medicinal chemistry, computational biology, clinical pharmacology and so on. Such therapies must not only overcome biological resistance but must also be deployable under real-world conditions: chemically stable, child-friendly, cost-effective and accessible to the communities that need them most. Achieving this vision will require balancing technological innovation with practical implementation and global equity. If these challenges can be met, the next generation of antimalarials may not simply sustain malaria control but help enable humanity’s long-standing goal of malaria elimination.

## Pending issues

1. **How can we identify or design molecules that access unexplored chemical space?** The scarcity of diverse chemical matter with the desired activity profiles remains a major constraint in antimalarial drug discovery, limiting the identification and exploitation of new therapeutic pathways.
2. **How can we design antimalarials that stay ahead of resistance?** Emphasis is needed on identifying druggable and resistance-refractory targets, predicting resistance trajectories early in development, and designing rational combinations that constrain parasite evolution.
3. **How can we better target the entire** ***Plasmodium*** **life cycle?** Improved experimental models and scalable screening assays for liver, sexual and mosquito stages are needed to deliver antimalarials that combine treatment with relapse prevention, chemoprotection and transmission blocking.
4. **Which emerging approaches will translate into deployable medicines?** Long-acting agents, new therapeutic modalities and drug repurposing offer considerable promise, but their clinical value will depend on achieving appropriate safety, pharmacokinetics and manufacturability.
5. **Can AI deliver measurable improvements in antimalarial drug development?** Progress will require high-quality and representative datasets, equitable data-sharing frameworks, prospective validation of predictions and integration of computational approaches with experimental biology.
6. **How can innovation translate into real-world impact?** Clinical development must incorporate early PK/PD and dose optimisation in children, pregnant women and diverse endemic populations. Public–private partnerships, interdisciplinary collaboration and leadership from malaria-endemic countries will be essential to ensure that next-generation therapies are effective, accessible and affordable.

## Supplementary information

Peer Review File

## Glossary

**ACTs (Artemisinin-based combination therapies)**: Combination treatments containing an artemisinin derivative and a partner drug that are the current standard of care for uncomplicated malaria.
**Functional genomics**: A set of experimental approaches used to determine gene function and identify genes essential for parasite survival, growth or drug susceptibility.
**Pharmacokinetics (PK)**: The processes governing the absorption, distribution, metabolism and excretion of a drug. Pharmacokinetic properties determine the onset, magnitude and duration of drug exposure, which influence treatment efficacy, dosing schedules and duration of protection.
**Pharmacodynamics (PD)**: The study of the biological effects of a drug on its target and the relationship between drug concentration and therapeutic activity.
**Chemical matter**: Bioactive compounds and their associated chemical series with desirable biological and physicochemical properties that can serve as starting points for drug discovery through medicinal chemistry advancement. High-quality chemical matter combines potency, selectivity and suitable pharmacological properties for further optimisation into clinical candidates.
**MoA (Mechanism of action)**: The molecular and cellular processes through which a drug produces its biological effect. Understanding the mechanism of action of a drug involves identifying its target(s), the pathways it perturbs and the downstream consequences that lead to parasite inhibition or death.
**Prophylaxis**: The use of interventions, including medicines, to prevent infection or disease before symptoms occur.
**ABS (Asexual blood stages)**: The disease-causing stages of *Plasmodium* that replicate within human erythrocytes.
**TCPs (Target candidate profiles)**: A framework defining the desired characteristics of a potential future antimalarial medicine, such as efficacy, safety, duration of action and stage-specificity.
**HTS (High-throughput screening)**: An automated approach for testing large numbers of compounds against a biological target or to identify a desirable phenotype.
**IVEWGA (In vitro evolution and whole-genome analysis)**: A target-identification method in which parasites evolved under drug pressure and resistance-associated genetic changes are identified by genome sequencing.
**Haemozoin formation inhibition**: A mode of action in which drugs block the detoxification of haem released during haemoglobin digestion, leading to parasite death.
**Chemoproteomics**: An experimental approach that combines small-molecule probes with proteomics to identify the protein targets and cellular interactions of bioactive compounds.
**MS-CETSA (Mass spectrometry-based cellular thermal shift assay)**: A proteome-wide method that detects drug–protein interactions by measuring changes in protein thermal stability after compound treatment.
**Biochemical assays**: In vitro experiments that measure the activity or inhibition of purified proteins, enzymes or molecular pathways. These assays complement phenotypic screening by providing evidence of target engagement and selectivity and can guide lead optimisation.
**Chemoprevention**: The administration of drugs to prevent malaria infection or disease in at-risk individuals.
**Phenotypic screening**: A drug-discovery approach that identifies compounds based on their effects on cells or organisms without requiring prior knowledge of the target or mechanism of action.
**Transmission blocking**: Preventing the spread of malaria parasites from humans to mosquitoes, thereby interrupting transmission. Unlike therapies that primarily treat disease-causing blood-stage infections, transmission-blocking interventions help reduce onward spread and support malaria elimination efforts.
**Radical cure**: Complete elimination of all parasite stages, including dormant liver forms that can cause relapse.
**Egress**: The active process by which mature malaria parasites exit infected host red blood cells to initiate a new replication cycle.
**Druggability**: The potential of a biological target to be modulated by a therapeutic agent with sufficient potency, selectivity, and safety to support drug development. This is determined by factors including target essentiality, accessibility, tractability to chemical modulation and the likelihood of resistance emergence.
**Irresistible (non-mutable)**: Describes a target or pathway for which the evolution of drug resistance is difficult. Non-mutable pathways are highly constrained and cannot easily acquire resistance-conferring mutations without compromising parasite fitness.
**Overexpression library**: A collection of genetically modified parasite lines engineered to overexpress individual genes, enabling systematic investigation of gene function, drug susceptibility and target identification. Increased expression of a drug target can confer reduced compound sensitivity, providing evidence for target engagement and mechanism of action.
**Transcriptome**: The complete set of RNA molecules expressed by a cell under defined conditions. The transcriptome is perturbed by drug treatment allowing mechanism of action to be inferred from observed changes.
**HDT (Host-directed therapy)**: A therapeutic strategy that targets host biological pathways rather than parasite targets to prevent or treat infection.
**TCIs (Targeted covalent inhibitors)**: Small molecules that form a selective, often irreversible covalent bond with their target protein to achieve prolonged inhibition.
**LAIs (Long-acting injectables)**: Formulations designed to slowly release drug over weeks or months after a single injection.
**Chimeric molecules**: Engineered compounds that combine two or more pharmacologically active components within a single chemical entity to simultaneously modulate multiple targets or biological processes.
**Target deconvolution**: The process of identifying the molecular target(s) and biological pathways responsible for the activity of a bioactive compound. Target deconvolution integrates genetic, chemical biology and proteomic approaches to link a phenotypic effect to its underlying mechanism of action.
**Optional extra terms:**** Multi-target**: A therapeutic strategy in which a single compound or combination affects multiple biological targets or pathways, also known as polypharmacology.
**Multi-modal models**: Artificial intelligence models capable of integrating and analysing multiple data types, such as text, images, genomic data and chemical structures.
**Non-mutable pathways**: Biological pathways that are highly constrained and cannot easily acquire resistance-conferring mutations without compromising parasite fitness.
**ABPP (Activity-based protein profiling)**: A chemoproteomic technique that uses reactive probes to identify functional or drug-engaged proteins in cells.
**Pharmacovigilance**: The monitoring and evaluation of drug safety after clinical use or market approval.
**Transgenic**: Referring to an organism that has been genetically modified to contain foreign or engineered DNA.
**Kinobead studies**: Chemical proteomics experiments that use kinase-binding affinity matrices to identify kinase targets and off-target interactions of small molecules.
**Chemically validated**: Describes a biological target for which pharmacological modulation by small molecules has demonstrated the expected biological effect.
**LLMs (Large-language models)**: Artificial intelligence systems trained on large text datasets that can generate, summarise or analyse scientific information.

## References

[CR2] Adam A, Besson D, Bryant R, Rees S, Willis PA, Burrows JN, Hooft van Huisjduijnen R, Laleu B, Norton L, Canan S et al (2024) Global health priority box─proactive pandemic preparedness. ACS Infect Dis 10:4030–4039 10.1021/acsinfecdis.4c00700 PMC11650653 39488746

[CR3] Adams KA, K, Fromer J, Coley CW (2025) ShEPhERD: diffusing shape, electrostatics, and pharmacophores for bioisosteric drug design. Preprint at arXiv:2411.04130

[CR4] Aleshnick M, Florez-Cuadros M, Martinson T, Wilder BK (2022) Monoclonal antibodies for malaria prevention. Mol Ther 30:1810–1821 10.1016/j.ymthe.2022.04.001 PMC8979832 35395399

[CR5] Aliper A, Kudrin R, Polykovskiy D, Kamya P, Tutubalina E, Chen S, Ren F, Zhavoronkov A (2023) Prediction of clinical trials outcomes based on target choice and clinical trial design with multi-modal artificial intelligence. Clin Pharm Ther 114:972–980 10.1002/cpt.3008 37483175

[CR6] Angrisano F, Tan YH, Sturm A, McFadden GI, Baum J (2012) Malaria parasite colonisation of the mosquito midgut—placing the *Plasmodium* ookinete centre stage. Int J Parasitol 42:519–527 10.1016/j.ijpara.2012.02.004 22406332

[CR7] Anthony MP, Burrows JN, Duparc S, Moehrle JJ, Wells TN (2012) The global pipeline of new medicines for the control and elimination of malaria. Malar J 11:316 10.1186/1475-2875-11-316 PMC3472257 22958514

[CR8] Antonova-Koch Y, Meister S, Abraham M, Luth MR, Ottilie S, Lukens AK, Sakata-Kato T, Vanaerschot M, Owen E, Jado JC et al (2018) Open-source discovery of chemical leads for next-generation chemoprotective antimalarials. Science 362:eaat9446 10.1126/science.aat9446 PMC6516198 30523084

[CR9] Arendse LB, Murithi JM, Qahash T, Pasaje CFA, Godoy LC, Dey S, Gibhard L, Ghidelli-Disse S, Drewes G, Bantscheff M et al (2022) The anticancer human mTOR inhibitor sapanisertib potently inhibits multiple *Plasmodium* kinases and life cycle stages. Sci Transl Med 14:eabo7219 10.1126/scitranslmed.abo7219 PMC9951552 36260689

[CR10] Arendse LB, Wyllie S, Chibale K, Gilbert IH (2021) Plasmodium kinases as potential drug targets for malaria: challenges and opportunities. ACS Infect Dis 7:518–534 10.1021/acsinfecdis.0c00724 PMC7961706 33590753

[CR11] Askin S, Burkhalter D, Calado G, El Dakrouni S (2023) Artificial Intelligence Applied to clinical trials: opportunities and challenges. Health Technol 13:203–213 10.1007/s12553-023-00738-2 PMC9974218 36923325

[CR12] Baker DA, Stewart LB, Large JM, Bowyer PW, Ansell KH, Jimenez-Diaz MB, El Bakkouri M, Birchall K, Dechering KJ, Bouloc NS et al (2017) A potent series targeting the malarial cGMP-dependent protein kinase clears infection and blocks transmission. Nat Commun 8:430 10.1038/s41467-017-00572-x PMC5585294 28874661

[CR13] Baldwin J, Michnoff CH, Malmquist NA, White J, Roth MG, Rathod PK, Phillips MA (2005) High-throughput screening for potent and selective inhibitors of *Plasmodium falciparum* dihydroorotate dehydrogenase. J Biol Chem 280:21847–21853 10.1074/jbc.M501100200 15795226

[CR14] Barnes KI, Little F, Smith PJ, Evans A, Watkins WM, White NJ (2006) Sulfadoxine-pyrimethamine pharmacokinetics in malaria: pediatric dosing implications. Clin Pharm Ther 80:582–596 10.1016/j.clpt.2006.08.016 17178260

[CR15] Beck JT, Rammage M, Jackson GP, Preininger AM, Dankwa-Mullan I, Roebuck MC, Torres A, Holtzen H, Coverdill SE, Williamson MP et al (2020) Artificial intelligence tool for optimizing eligibility screening for clinical trials in a large community cancer center. JCO Clin Cancer Inf 4:50–59 10.1200/CCI.19.00079 31977254

[CR16] Begolo D, Erben E, Clayton C (2014) Drug target identification using a trypanosome overexpression library. Antimicrob Agents Chemother 58:6260–6264 10.1128/AAC.03338-14 PMC4187942 25049244

[CR17] Benns HJ, Storch M, Falco JA, Fisher FR, Tamaki F, Alves E, Wincott CJ, Milne R, Wiedemar N, Craven G et al (2022) CRISPR-based oligo recombineering prioritizes apicomplexan cysteines for drug discovery. Nat Microbiol 7:1891–1905 10.1038/s41564-022-01249-y PMC9613468 36266336

[CR18] Birkholtz LM, Olivier T, Welcome T, Strauss E (2026) Targeted protein degradation as a novel therapeutic strategy against infectious diseases. Curr Opin Chem Biol 91:102655 10.1016/j.cbpa.2026.102655 41759247

[CR19] Borba JVB, de Azevedo BR, Ferreira LA, Rimoldi A, Salazar Alvarez LC, Calit J, Bargieri DY, Costa FTM, Andrade CH (2023) Transcriptomics-guided in silico drug repurposing: identifying new candidates with dual-stage antiplasmodial activity. ACS Omega 8:34084–34090 10.1021/acsomega.3c05138 PMC10515587 37744849

[CR20] Bosc N, Felix E, Arcila R, Mendez D, Saunders MR, Green DVS, Ochoada J, Shelat AA, Martin EJ, Iyer P et al (2021) MAIP: a web service for predicting blood-stage malaria inhibitors. J Cheminform 13:13 10.1186/s13321-021-00487-2 PMC7898753 33618772

[CR21] Bousema T, Drakeley C (2011) Epidemiology and infectivity of *Plasmodium falciparum* and *Plasmodium vivax* gametocytes in relation to malaria control and elimination. Clin Microbiol Rev 24:377–410 10.1128/CMR.00051-10 PMC3122489 21482730

[CR22] Bower-Lepts C, Rawat M, Keith WC, Girling G, Luth MR, Carolino K, Boroviak K, Coyle R, Schwach F, Smidt C et al (2024) A novel chemogenomic screening platform for scalable antimalarial drug target identification. Preprint at bioRxiv 10.1101/2024.08.27.609875

[CR23] Brancucci NMB, Gumpp C, van Gemert G-J, Yu X, Passecker A, Nardella F, Thommen BT, Chambon M, Turcatti G, Halby L et al (2025) An all-in-one pipeline for the in vitro discovery and in vivo testing of *Plasmodium falciparum* malaria transmission blocking drugs. Nat Commun 16:6884 10.1038/s41467-025-62014-3 PMC12297408 40715087

[CR24] Braunack-Mayer L, Malinga J, Nekkab N, Kelly SL, Mohrle JJ, Penny MA (2026) Combining seasonal malaria chemoprevention with novel therapeutics for malaria prevention: a mathematical modelling study. PLoS Comput Biol 22:e1014021 10.1371/journal.pcbi.1014021 PMC12962535 41746945

[CR25] Brettell SB, Cann G, Begen A, Sharma S, Mahindra A, Carruthers LV, Milligan G, Clarke DJ, Tobin AB, Jamieson AG (2025) Lysine targeting covalent inhibitors of malarial kinase PfCLK3. RSC Med Chem 16:3530–3540 10.1039/d5md00335k PMC12164071 40521344

[CR26] Brettell SB, Janha O, Begen A, Cann G, Sharma S, Olaniyan N, Yelland T, Hole AJ, Alam B, Mayville E et al (2024) Targeting PfCLK3 with covalent inhibitors: a novel strategy for malaria treatment. J Med Chem 67:18895–18910 10.1021/acs.jmedchem.4c01300 PMC11571108 39441986

[CR27] Burrows JN, Duparc S, Gutteridge WE, Hooft van Huijsduijnen R, Kaszubska W, Macintyre F, Mazzuri S, Möhrle JJ, Wells TNC (2017) New developments in anti-malarial target candidate and product profiles. Malar J 16:26 10.1186/s12936-016-1675-x PMC5237200 28086874

[CR28] Button-Simons KA, Kumar S, Carmago N, Haile MT, Jett C, Checkley LA, Kennedy SY, Pinapati RS, Shoue DA, McDew-White M et al (2021) The power and promise of genetic mapping from *Plasmodium falciparum* crosses utilizing human liver-chimeric mice. Commun Biol 4:734 10.1038/s42003-021-02210-1 PMC8203791 34127785

[CR29] Calit J, Araújo Jessica E, Deng B, Miura K, Gaitán Xiomara A, Araújo Maisa da S, Medeiros Jansen F, Long Carole A, Simeonov A, Eastman Richard T et al (2023) Novel transmission-blocking antimalarials identified by high-throughput screening of *Plasmodium berghei* Ookluc. Antimicrob Agents Chemother 67:e01465–01422 10.1128/aac.01465-22 PMC10112123 36856421

[CR30] Carolino K, Winzeler EA (2020) The antimalarial resistome – finding new drug targets and their modes of action. Curr Opin Microbiol 57:49–55 10.1016/j.mib.2020.06.004 PMC7763834 32682267

[CR31] Challis MP, Devine SM, Creek DJ (2022) Current and emerging target identification methods for novel antimalarials. Int J Parasitol Drugs Drug Resist 20:135–144 10.1016/j.ijpddr.2022.11.001 PMC9771836 36410177

[CR32] Chandrasekaran SN, Ceulemans H, Boyd JD, Carpenter AE (2021) Image-based profiling for drug discovery: due for a machine-learning upgrade? Nat Rev Drug Discov 20:145–159 10.1038/s41573-020-00117-w PMC7754181 33353986

[CR33] Chesbrough H, Chen EL (2013) Recovering abandoned compounds through expanded external IP licensing. Calif Manag Rev 55:83–101

[CR34] Chien HD, Pantaleo A, Kesely KR, Noomuna P, Putt KS, Tuan TA, Low PS, Turrini FM (2021) Imatinib augments standard malaria combination therapy without added toxicity. J Exp Med 218:e20210724 10.1084/jem.20210724 PMC8404470 34436509

[CR35] Choudhuri S, Ghosh B (2024) Computational approach for decoding malaria drug targets from single-cell transcriptomics and finding potential drug molecule. Sci Rep 14:24064 10.1038/s41598-024-72427-7 PMC11473826 39402081

[CR1] ClinicalTrials.gov. Safety and PK of MMV371 LAI in healthy adults and adolescents in Rwanda. https://clinicaltrials.gov/study/NCT07548021

[CR36] Cowell AN, Winzeler EA (2019) Advances in omics-based methods to identify novel targets for malaria and other parasitic protozoan infections. Genome Med 11:63 10.1186/s13073-019-0673-3 PMC6805675 31640748

[CR37] Crowther GJ, Hillesland HK, Keyloun KR, Reid MC, Lafuente-Monasterio MJ, Ghidelli-Disse S, Leonard SE, He P, Jones JC, Krahn MM et al (2016) Biochemical screening of five protein kinases from *Plasmodium falciparum* against 14,000 cell-active compounds. PLoS ONE 11:e0149996 10.1371/journal.pone.0149996 PMC4774911 26934697

[CR38] Crutcher JM, Hoffman SL (1996) Malaria. In: Baron S (ed) Medical microbiology. University of Texas Medical Branch, Galveston 21413352

[CR39] D’Alessandro S, Camarda G, Corbett Y, Siciliano G, Parapini S, Cevenini L, Michelini E, Roda A, Leroy D, Taramelli D et al (2016) A chemical susceptibility profile of the *Plasmodium falciparum* transmission stages by complementary cell-based gametocyte assays. J Antimicrob Chemother 71:1148–1158 10.1093/jac/dkv493 26888912

[CR40] de Sousa ACC, Combrinck JM, Maepa K, Egan TJ (2020) Virtual screening as a tool to discover new β-haematin inhibitors with activity against malaria parasites. Sci Rep 10:3374 10.1038/s41598-020-60221-0 PMC7042288 32099045

[CR41] Delves MJ, Miguel-Blanco C, Matthews H, Molina I, Ruecker A, Yahiya S, Straschil U, Abraham M, León ML, Fischer OJ et al (2018) A high throughput screen for next-generation leads targeting malaria parasite transmission. Nat Commun 9:3805 10.1038/s41467-018-05777-2 PMC6143625 30228275

[CR42] Demarta-Gatsi C, Donini C, Duffy J, Sadler C, Stewart J, Barber JA, Tornesi B (2022) Malarial PI4K inhibitor induced diaphragmatic hernias in rat: potential link with mammalian kinase inhibition. Birth Defects Res 114:487–498 10.1002/bdr2.2012 PMC9321963 35416431

[CR43] Dhillon S (2023) Adagrasib: first approval. Drugs 83:275–285 10.1007/s40265-023-01839-y 36763320

[CR44] Douglas AD, Baldeviano GC, Jin J, Miura K, Diouf A, Zenonos ZA, Ventocilla JA, Silk SE, Marshall JM, Alanine DGW et al (2019) A defined mechanistic correlate of protection against *Plasmodium falciparum* malaria in non-human primates. Nat Commun 10:1953 10.1038/s41467-019-09894-4 PMC6486575 31028254

[CR45] Duffey M, Blasco B, Burrows JN, Wells TNC, Fidock DA, Leroy D (2021) Assessing risks of *Plasmodium falciparum* resistance to select next-generation antimalarials. Trends Parasitol 37:709–721 10.1016/j.pt.2021.04.006 PMC8282644 34001441

[CR46] Dziekan JM, Wirjanata G, Dai L, Go KD, Yu H, Lim YT, Chen L, Wang LC, Puspita B, Prabhu N et al (2020) Cellular thermal shift assay for the identification of drug–target interactions in the *Plasmodium falciparum* proteome. Nat Protoc 15:1881–1921 10.1038/s41596-020-0310-z 32341577

[CR47] Dziekan JM, Yu H, Chen D, Dai L, Wirjanata G, Larsson A, Prabhu N, Sobota RM, Bozdech Z, Nordlund P (2019) Identifying purine nucleoside phosphorylase as the target of quinine using cellular thermal shift assay. Sci Transl Med 11:eaau3174 10.1126/scitranslmed.aau3174 30602534

[CR48] Ebel ER, Kuypers FA, Lin C, Petrov DA, Egan ES (2021) Common host variation drives malaria parasite fitness in healthy human red cells. eLife 10:e69808 10.7554/eLife.69808 PMC8497061 34553687

[CR49] El Gaaloul M, Tchouatieu AM, Kayentao K, Campo B, Buffet B, Ramachandruni H, Ndiaye JL, Wells TNC, Audibert C, Achan J et al (2024) Chemoprevention of malaria with long-acting oral and injectable drugs: an updated target product profile. Malar J 23:315 10.1186/s12936-024-05128-1 PMC11490162 39425110

[CR50] Espinoza JL (2025) Candidate drug repurposing for malaria: perspectives for optimising clinical trials. Lancet Region Health Am 41:100939 10.1016/j.lana.2024.100939 PMC11613157 39628989

[CR51] Ferreira FJN, Carneiro AS (2025) AI-driven drug discovery: a comprehensive review. ACS Omega 10:23889–23903 10.1021/acsomega.5c00549 PMC12177741 40547666

[CR52] Flannery EL, Chatterjee AK, Winzeler EA (2013) Antimalarial drug discovery - approaches and progress towards new medicines. Nat Rev Microbiol 11:849–862 10.1038/nrmicro3138 PMC3941073 24217412

[CR53] Forte B, Ottilie S, Plater A, Campo B, Dechering KJ, Gamo FJ, Goldberg DE, Istvan ES, Lee M, Lukens AK et al (2021a) Prioritization of molecular targets for antimalarial drug discovery. ACS Infect Dis 7:2764–2776 10.1021/acsinfecdis.1c00322 PMC8608365 34523908

[CR54] Forte B, Ottilie S, Plater A, Campo B, Dechering KJ, Gamo FJ, Goldberg DE, Istvan ES, Lee M, Lukens AK et al (2021b) Prioritization of molecular targets for antimalarial drug discovery. ACS Infect Dis 7:2764–2776 10.1021/acsinfecdis.1c00322 PMC8608365 34523908

[CR55] Francis SE, Sullivan DJ Jr., Goldberg DE (1997) Hemoglobin metabolism in the malaria parasite Plasmodium falciparum. Annu Rev Microbiol 51:97–123 10.1146/annurev.micro.51.1.97 9343345

[CR56] Frevert U (2004) Sneaking in through the back entrance: the biology of malaria liver stages. Trends Parasitol 20:417–424 10.1016/j.pt.2004.07.007 15324732

[CR57] Gachuhi S, Kamunya S, Fienberg S, Wambua L, Salomane N, Mayoka G, Taylor D, Coertzen D, van der Watt M, Reader J et al (2025) Medicinal chemistry progression of sapanisertib, the anticancer and dual *Plasmodium* phosphatidylinositol 4-kinase beta and cGMP-dependent protein kinase inhibitor, for malaria. J Med Chem 68:10757–10770 10.1021/acs.jmedchem.4c02799 PMC12169674 40377111

[CR58] Gamo FJ, Sanz LM, Vidal J, de Cozar C, Alvarez E, Lavandera JL, Vanderwall DE, Green DV, Kumar V, Hasan S et al (2010) Thousands of chemical starting points for antimalarial lead identification. Nature 465:305–310 10.1038/nature09107 20485427

[CR59] Gazanion É, Fernández-Prada C, Papadopoulou B, Leprohon P, Ouellette M (2016) Cos-Seq for high-throughput identification of drug target and resistance mechanisms in the protozoan parasite *Leishmania*. Proc Natl Acad Sci USA 113:E3012–E3021 10.1073/pnas.1520693113 PMC4889358 27162331

[CR60] Godara P, Reddy KS, Sahu W, Naik B, Srivastava V, Das R, Mahor A, Kumar P, Giri R, Anirudh J et al (2024) Structure-based virtual screening against multiple *Plasmodium falciparum* kinases reveals antimalarial compounds. Mol Divers 28:3661–3681 10.1007/s11030-023-10770-z 38127294

[CR61] Godinez-Macias KP, Chen D, Wallis JL, Siegel MG, Adam A, Bopp S, Carolino K, Coulson LB, Durst G, Thathy V et al (2025a) Revisiting the *Plasmodium falciparum* druggable genome using predicted structures and data mining. npj Drug Discov 2:3 10.1038/s44386-025-00006-5 PMC11892419 40066064

[CR62] Godinez-Macias KP, Chen D, Wallis JL, Siegel MG, Adam A, Bopp S, Carolino K, Coulson LB, Durst G, Thathy V et al (2025b) Revisiting the *Plasmodium falciparum* druggable genome using predicted structures and data mining. NPJ Drug Discov 2:3 10.1038/s44386-025-00006-5 PMC11892419 40066064

[CR63] Gomez F, Hirbo J, Tishkoff SA (2014) Genetic variation and adaptation in Africa: implications for human evolution and disease. Cold Spring Harb Perspect Biol 6:a008524 10.1101/cshperspect.a008524 PMC4067985 24984772

[CR64] Greenwood B, Cairns M, Chaponda M, Chico RM, Dicko A, Ouedraogo JB, Phiri KS, Ter Kuile FO, Chandramohan D (2021) Combining malaria vaccination with chemoprevention: a promising new approach to malaria control. Malar J 20:361 10.1186/s12936-021-03888-8 PMC8419817 34488784

[CR65] Guerra F, Winzeler EA (2022) New targets for antimalarial drug discovery. Curr Opin Microbiol 70:102220 10.1016/j.mib.2022.102220 PMC9934905 36228458

[CR66] Guiguemde WA, Shelat AA, Bouck D, Duffy S, Crowther GJ, Davis PH, Smithson DC, Connelly M, Clark J, Zhu F et al (2010) Chemical genetics of Plasmodium falciparum. Nature 465:311–315 10.1038/nature09099 PMC2874979 20485428

[CR67] Gupta AK, Eliasen AM, Andahazy W, Zhou F, Henson K, Chi V, Woods AK, Joseph SB, Kuhen KL, Wisler J et al (2024) A prodrug strategy to reposition atovaquone et as a long-acting injectable for malaria chemoprotection. Preprint at bioRxiv 10.1101/2024.02.08.579395

[CR68] Ha SV, Jaensch S, Kańduła MM, Herman D, Czodrowski P, Ceulemans H (2025) Cross modality learning of cell painting and transcriptomics data improves mechanism of action clustering and bioactivity modelling. Sci Rep 15:23010 10.1038/s41598-025-05914-0 PMC12216865 40596104

[CR69] Haga SB (2024) Artificial intelligence, medications, pharmacogenomics, and ethics. Pharmacogenomics 25:611–622 10.1080/14622416.2024.2428587 PMC11703462 39545629

[CR70] Hanefeld J (2014) The global fund to fight AIDS, tuberculosis and malaria: 10 years on. Clin Med 14:54–57 10.7861/clinmedicine.14-1-54 PMC5873622 24532746

[CR71] Hooft van Huijsduijnen R, Wells T, Tanner M, Wittlin S (2019) Two successful decades of Swiss collaborations to develop new anti-malarials. Malar J 18:94 10.1186/s12936-019-2728-8 PMC6431002 30902051

[CR72] Hovlid ML, Winzeler EA (2016) Phenotypic screens in antimalarial drug discovery. Trends Parasitol 32:697–707 10.1016/j.pt.2016.04.014 PMC5007148 27247245

[CR73] Ishii R, Sakura T, Hong J, Yasuo K, Hattori K, Willis PA, Kato T, Inaoka DK (2025) Efficient identification of antimalarials with novel mechanism of action via multi-sample transcriptomic profiling. Preprint at bioRxiv 10.1101/2025.02.27.640686

[CR74] Ivanov A, Getova-Kolarova V, Getov I (2024) Research and analysis of regulatory framework and harmonisation of repurposing. Pharmacia 71:1–7

[CR75] Jayatunga MKP, Ayers M, Bruens L, Jayanth D, Meier C (2024) How successful are AI-discovered drugs in clinical trials? A first analysis and emerging lessons. Drug Discov Today 29:104009 10.1016/j.drudis.2024.104009 38692505

[CR76] Kamya P, Ozerov IV, Pun FW, Tretina K, Fokina T, Chen S, Naumov V, Long X, Lin S, Korzinkin M et al (2024) PandaOmics: an AI-driven platform for therapeutic target and biomarker discovery. J Chem Inf Model 64:3961–3969 10.1021/acs.jcim.3c01619 PMC11134400 38404138

[CR77] Kanai M, Mok S, Yeo T, Shears MJ, Jeon JH, Narwal SK, Ross LS, Wiradiputri K, Haile MT, Tripathi AK et al (2026) Identification of genetic markers of quinine partial resistance in *Plasmodium falciparum*. Nat Microbiol 11:2213–2231 10.1038/s41564-026-02410-7 PMC13423875 42410209

[CR78] Kane J, Li X, Kumar S, Button-Simons KA, Vendrely Brenneman KM, Dahlhoff H, Sievert MAC, Checkley LA, Shoue DA, Singh PP et al (2024) A *Plasmodium falciparum* genetic cross reveals the contributions of pfcrt and plasmepsin II/III to piperaquine drug resistance. mBio 15:e0080524 10.1128/mbio.00805-24 PMC11253641 38912775

[CR79] Karunakaran D, Mutyam SK, Fu M, Chen J, Pham KHN, Pou S, Winter RW, Nilsen A, Dodean RA, Smilkstein MJ et al (2024) Long-acting intramuscular injections of ELQ-331, an antimalarial agent. Eur J Pharm Sci 198:106795 10.1016/j.ejps.2024.106795 PMC11160314 38729224

[CR80] Kesely KR, Pantaleo A, Turrini FM, Olupot-Olupot P, Low PS (2016) Inhibition of an erythrocyte tyrosine kinase with imatinib prevents *Plasmodium falciparum* egress and terminates parasitemia. PLoS ONE 11:e0164895 10.1371/journal.pone.0164895 PMC5074466 27768734

[CR81] Kester KE, Gray Heppner D, Moris P, Ofori-Anyinam O, Krzych U, Tornieporth N, McKinney D, Delchambre M, Ockenhouse CF, Voss G et al (2014) Sequential Phase 1 and Phase 2 randomized, controlled trials of the safety, immunogenicity and efficacy of combined pre-erythrocytic vaccine antigens RTS,S and TRAP formulated with AS02 adjuvant system in healthy, malaria naïve adults. Vaccine 32:6683–6691 10.1016/j.vaccine.2014.06.033 24950358

[CR82] Khan S (2016) Recent advances in the biology and drug targeting of malaria parasite aminoacyl-tRNA synthetases. Malar J 15:203 10.1186/s12936-016-1247-0 PMC4828885 27068331

[CR83] Knockaert M, Gray N, Damiens E, Chang YT, Grellier P, Grant K, Fergusson D, Mottram J, Soete M, Dubremetz JF et al (2000) Intracellular targets of cyclin-dependent kinase inhibitors: identification by affinity chromatography using immobilised inhibitors. Chem Biol 7:411–422 10.1016/s1074-5521(00)00124-1 10873834

[CR84] Krishnamurthy N, Grimshaw AA, Axson SA, Choe SH, Miller JE (2022) Drug repurposing: a systematic review on root causes, barriers and facilitators. BMC Health Serv Res 22:970 10.1186/s12913-022-08272-z PMC9336118 35906687

[CR85] Langeveld H, Maepa K, Maree M, Thibaud JL, Salomane N, Bridgwater R, Famodimu MT, Godoy LC, Pasaje CFA, Boonyalai N et al (2025) Targeting aurora kinases as essential cell-cycle regulators to deliver multi-stage antimalarials against *Plasmodium Falciparum*. Angew Chem Int Ed Engl 64:e202518493 10.1002/anie.202518493 PMC12707362 41131905

[CR86] Lee MCS, Lindner SE, Lopez-Rubio JJ, Llinas M (2019) Cutting back malaria: CRISPR/Cas9 genome editing of *Plasmodium*. Brief Funct Genom 18:281–289 10.1093/bfgp/elz012 PMC6859820 31365053

[CR87] Leung H, Duan C, Gou W, Chen J, Xin Y, Zheng Z, Naumov V, Gennert D, Zhang M, Aliper A et al (2026) Advancing target discovery through disease-specific integration of multi-modal target identification models and comprehensive benchmarking system. Sci Rep 16:17018 10.1038/s41598-026-47765-3 PMC13230693 41968138

[CR88] Loeffler HH, He J, Tibo A, Janet JP, Voronov A, Mervin LH, Engkvist O (2024) Reinvent 4: modern AI-driven generative molecule design. J Cheminform 16:20 10.1186/s13321-024-00812-5 PMC10882833 38383444

[CR89] Lu K-Y, Mansfield CR, Fitzgerald MC, Derbyshire ER (2021a) Chemoproteomics for *Plasmodium* parasite drug target discovery. ChemBioChem 22:2591–2599 10.1002/cbic.202100155 PMC8373781 33999499

[CR90] Lu KY, Mansfield CR, Fitzgerald MC, Derbyshire ER (2021b) Chemoproteomics for *Plasmodium* parasite drug target discovery. Chembiochem 22:2591–2599 10.1002/cbic.202100155 PMC8373781 33999499

[CR91] Lucantoni L, Fidock DavidA, Avery VickyM (2016) Luciferase-based, high-throughput assay for screening and profiling transmission-blocking compounds against *Plasmodium falciparum* gametocytes. Antimicrob Agents Chemother 60:2097–2107 10.1128/AAC.01949-15 PMC4808229 26787698

[CR92] Luth MR, Godinez-Macias KP, Chen D, Okombo J, Thathy V, Cheng X, Daggupati S, Davies H, Dhingra SK, Economy JM et al (2024) Systematic in vitro evolution in *Plasmodium falciparum* reveals key determinants of drug resistance. Science 386:eadk9893 10.1126/science.adk9893 PMC11809290 39607932

[CR93] Luth MR, Gupta P, Ottilie S, Winzeler EA (2018) Using in vitro evolution and whole genome analysis to discover next generation targets for antimalarial drug discovery. ACS Infect Dis 4:301–314 10.1021/acsinfecdis.7b00276 PMC5848146 29451780

[CR94] Macintyre F, Ramachandruni H, Burrows JN, Holm R, Thomas A, Möhrle JJ, Duparc S, Hooft van Huijsduijnen R, Greenwood B, Gutteridge WE et al (2018) Injectable anti-malarials revisited: discovery and development of new agents to protect against malaria. Malar J 17:402 10.1186/s12936-018-2549-1 PMC6211409 30384848

[CR95] Maher SP, Bakowski MA, Vantaux A, Flannery EL, Andolina C, Gupta M, Antonova-Koch Y, Argomaniz M, Cabrera-Mora M, Campo B et al (2025) A drug repurposing approach reveals targetable epigenetic pathways in *Plasmodium vivax* hypnozoites. eLife 13:RP98221 10.7554/eLife.98221 PMC12483515 41025349

[CR96] Maher SP, Vantaux A, Chaumeau V, Chua ACY, Cooper CA, Andolina C, Péneau J, Rouillier M, Rizopoulos Z, Phal S et al (2021a) Probing the distinct chemosensitivity of *Plasmodium vivax* liver stage parasites and demonstration of 8-aminoquinoline radical cure activity in vitro. Sci Rep 11:19905 10.1038/s41598-021-99152-9 PMC8497498 34620901

[CR97] Maher SP, Vantaux A, Cooper CA, Chasen NM, Cheng WT, Joyner CJ, Manetsch R, Witkowski B, Kyle D (2021b) A phenotypic screen for the liver stages of *Plasmodium vivax*. Bio Protoc 11:e4253 10.21769/BioProtoc.4253 PMC8678555 35005096

[CR98] Manzoni G, Marinach C, Topçu S, Briquet S, Grand M, Tolle M, Gransagne M, Lescar J, Andolina C, Franetich J-F et al (2017) *Plasmodium* P36 determines host cell receptor usage during sporozoite invasion. eLife 6:e25903 10.7554/eLife.25903 PMC5470872 28506360

[CR99] Margaret AP, Pradipsinh KR (2010) *Plasmodium* dihydroorotate dehydrogenase: a promising target for novel anti-malarial chemotherapy. Infect Disord Drug Targets 10:226–239 10.2174/187152610791163336 PMC2883174 20334617

[CR100] McNamara CW, Lee MC, Lim CS, Lim SH, Roland J, Simon O, Yeung BK, Chatterjee AK, McCormack SL, Manary MJ et al (2013) Targeting *Plasmodium* PI(4)K to eliminate malaria. Nature 504:248–253 10.1038/nature12782 PMC3940870 24284631

[CR101] Miguel-Blanco C, Lelièvre J, Delves Michael J, Bardera Ana I, Presa Jesús L, López-Barragán María J, Ruecker A, Marques S, Sinden Robert E, Herreros E (2015) Imaging-based high-throughput screening assay to identify new molecules with transmission-blocking potential against *Plasmodium falciparum* female gamete formation. Antimicrob Agents Chemother 59:3298–3305 10.1128/AAC.04684-14 PMC4432159 25801574

[CR102] Mohammed M, Dziedziech A, Macedo D, Huppertz F, Veith Y, Postel Z, Christ E, Scheytt R, Slotte T, Henriksson J et al (2024) Single-cell transcriptomics reveal transcriptional programs underlying male and female cell fate during *Plasmodium falciparum* gametocytogenesis. Nat Commun 15:7177 10.1038/s41467-024-51201-3 PMC11347709 39187486

[CR103] Mohammed R, Asres MS, Gudina EK, Adissu W, Johnstone H, Marrast AC, Donini C, Duparc S, Yilma D (2023) Efficacy, safety, tolerability, and pharmacokinetics of MMV390048 in acute uncomplicated malaria. Am J Trop Med Hyg 108:81–84 10.4269/ajtmh.22-0567 PMC9833083 36509063

[CR104] Moons P, Dou B, Desmedt CP (2025) Google’s AI co-scientist and OpenAI’s deep research: new partners in health research? Eur J Cardiovasc Nurs 24:800–807 10.1093/eurjcn/zvaf066 40678974

[CR105] Napoli JA, Reutlinger M, Brandl P, Wang W, Hert J, Desai P (2025) Multitask deep learning models of combined industrial absorption, distribution, metabolism, and excretion datasets to improve generalization. Mol Pharm 22:1892–1900 10.1021/acs.molpharmaceut.4c01086 40053846

[CR106] Nasto B (2025) Non-profits wade in where for-profit life science investment fears to tread. Nat Biotechnol 43:1221–1226 10.1038/s41587-025-02752-3 40696190

[CR107] Ng CL, Fidock DA (2019) *Plasmodium falciparum* in vitro drug resistance selections and gene editing. Methods Mol Biol 2013:123–140 10.1007/978-1-4939-9550-9_9 PMC6756925 31267498

[CR108] Ng CL, Fidock DA, Bogyo M (2017) Protein degradation systems as antimalarial therapeutic targets. Trends Parasitol 33:731–743 10.1016/j.pt.2017.05.009 PMC5656264 28688800

[CR109] Nguyen G-B, Cooper CA, McWhorter O, Sharma R, Elliot A, Ruberto A, Freitas R, Pathak AK, Kyle DE, Maher SP (2024) Screening the global health priority box against *Plasmodium berghei* liver stage parasites using an inexpensive luciferase detection protocol. Malar J 23:357 10.1186/s12936-024-05155-y PMC11585928 39580415

[CR110] Nguyen TD, Gao B, Amaratunga C, Dhorda M, Tran TN-A, White NJ, Dondorp AM, Boni MF, Aguas R (2023) Preventing antimalarial drug resistance with triple artemisinin-based combination therapies. Nat Commun 14:4568 10.1038/s41467-023-39914-3 PMC10387089 37516752

[CR111] Odah M (2025) Artificial intelligence meets drug discovery: a systematic review on AI-powered target identification and molecular design. 10.20944/preprints202503.0912.v1

[CR112] Okeke CJ, Ksouri A (2026) The role of precision medicine in malaria: an innovative treatment approach. In: Radouani F, Zass L, Fadlelmola FM (eds) Precision medicine in Africa: promises and opportunities. Springer Nature Singapore, pp 145–160

[CR113] Okombo J, Kanai M, Deni I, Fidock DA (2021) Genomic and genetic approaches to studying antimalarial drug resistance and *Plasmodium* Biology. Trends Parasitol 37:476–492 10.1016/j.pt.2021.02.007 PMC8162148 33715941

[CR114] Ouologuem DT, Dara A, Kone A, Ouattara A, Djimde AA (2023) *Plasmodium falciparum* development from gametocyte to oocyst: insight from functional studies. Microorganisms 11:1966 10.3390/microorganisms11081966 PMC10460021 37630530

[CR115] Painter HJ, Chung NC, Sebastian A, Albert I, Storey JD, Llinas M (2018) Genome-wide real-time in vivo transcriptional dynamics during Plasmodium falciparum blood-stage development. Nat Commun 9:2656 10.1038/s41467-018-04966-3 PMC6037754 29985403

[CR116] Paonessa G, Siciliano G, Graziani R, Lalli C, Cecchetti O, Alli C, La Valle R, Petrocchi A, Sferrazza A, Bisbocci M et al (2022) Gametocyte-specific and all-blood-stage transmission-blocking chemotypes discovered from high throughput screening on Plasmodium falciparum gametocytes. Commun Biol 5:547 10.1038/s42003-022-03510-w PMC9170688 35668202

[CR117] Paquet T, Le Manach C, Cabrera DG, Younis Y, Henrich PP, Abraham TS, Lee MCS, Basak R, Ghidelli-Disse S, Lafuente-Monasterio MJ et al (2017a) Antimalarial efficacy of MMV390048, an inhibitor of Plasmodium phosphatidylinositol 4-kinase. Sci Transl Med 9:eaad9735 10.1126/scitranslmed.aad9735 PMC5731459 28446690

[CR118] Paquet T, Le Manach C, Cabrera DG, Younis Y, Henrich PP, Abraham TS, Lee MCS, Basak R, Ghidelli-Disse S, Lafuente-Monasterio MJ et al (2017b) Antimalarial efficacy of MMV390048, an inhibitor of Plasmodium phosphatidylinositol 4-kinase. Sci Transl Med 9:eaad9735 10.1126/scitranslmed.aad9735 PMC5731459 28446690

[CR119] Pavadai E, El Mazouni F, Wittlin S, de Kock C, Phillips MA, Chibale K (2016) Identification of new human malaria parasite *Plasmodium falciparum* dihydroorotate dehydrogenase inhibitors by pharmacophore and structure-based virtual screening. J Chem Inf Model 56:548–562 10.1021/acs.jcim.5b00680 PMC4871132 26915022

[CR120] Pazicky S, Dziekan JM, Tjia S, Bopp S, Wirth D, Bozdech Z (2026) Thermal proteome profiling identifies new drug targets in *Plasmodium falciparum* parasites. Preprint at bioRxiv 10.64898/2026.01.30.70272442843592

[CR121] Penzo M, de las Heras-Dueña L, Mata-Cantero L, Diaz-Hernandez B, Vazquez-Muñiz M-J, Ghidelli-Disse S, Drewes G, Fernandez-Alvaro E, Baker DA (2019) High-throughput screening of the *Plasmodium falciparum* cGMP-dependent protein kinase identified a thiazole scaffold which kills erythrocytic and sexual stage parasites. Sci Rep 9:7005 10.1038/s41598-019-42801-x PMC6504873 31065005

[CR122] Phillips MA, Lotharius J, Marsh K, White J, Dayan A, White KL, Njoroge JW, El Mazouni F, Lao Y, Kokkonda S et al (2015) A long-duration dihydroorotate dehydrogenase inhibitor (DSM265) for prevention and treatment of malaria. Sci Transl Med 7:296ra111–296ra111 10.1126/scitranslmed.aaa6645 PMC4539048 26180101

[CR123] Plouffe David M, Wree M, Du Alan Y, Meister S, Li F, Patra K, Lubar A, Okitsu Shinji L, Flannery Erika L, Kato N et al (2016) High-throughput assay and discovery of small molecules that interrupt malaria transmission. Cell Host Microbe 19:114–126 10.1016/j.chom.2015.12.001 PMC4723716 26749441

[CR124] Plowe CV (2022) Malaria chemoprevention and drug resistance: a review of the literature and policy implications. Malar J 21:104 10.1186/s12936-022-04115-8 PMC8943514 35331231

[CR125] Probst AS, Paton DG, Appetecchia F, Bopp S, Adams KL, Rinvee TA, Pou S, Winter R, Du EW, Yahiya S et al (2025) In vivo screen of Plasmodium targets for mosquito-based malaria control. Nature 643:785–793 10.1038/s41586-025-09039-2 PMC12267055 40399670

[CR126] Pun FW, Ozerov IV, Zhavoronkov A (2023) AI-powered therapeutic target discovery. Trends Pharm Sci 44:561–572 10.1016/j.tips.2023.06.010 37479540

[CR127] Recino A, Rayner MLD, Rohn JL, Della Pasqua O, Recino A, Rayner MLD, Rohn JL, Carter B, Cupani A, Dahlmann-Noor A et al (2025) Therapeutic innovation in drug repurposing: challenges and opportunities. Drug Discov Today 30:104390 10.1016/j.drudis.2025.104390 40441598

[CR128] Redhi D, Mulubwa M, Mmonwa MM, Njoroge M, Krugmann L, Chibale K (2026) Repurposing drugs for malaria through a human dose prediction: a case study with berzosertib. Clin Pharmacol Ther. 10.1002/cpt.70303 PMC13339404 42080628

[CR129] Rogawski MA, Federoff HJ (2011) Disclosure of clinical trial results when product development is abandoned. Sci Transl Med 3:102cm129–102cm129 10.1126/scitranslmed.3002939 21957167

[CR130] Roth A, Maher SP, Conway AJ, Ubalee R, Chaumeau V, Andolina C, Kaba SA, Vantaux A, Bakowski MA, Thomson-Luque R et al (2018) A comprehensive model for assessment of liver stage therapies targeting *Plasmodium vivax* and *Plasmodium falciparum*. Nat Commun 9:1837 10.1038/s41467-018-04221-9 PMC5943321 29743474

[CR131] Singer M, Kanatani S, Castillo SG, Frischknecht F, Sinnis P (2024) The *Plasmodium* circumsporozoite protein. Trends Parasitol 40:1124–1134 10.1016/j.pt.2024.10.017 PMC12223538 39572325

[CR132] Singh J (2022) The ascension of targeted covalent inhibitors. J Med Chem 65:5886–5901 10.1021/acs.jmedchem.1c02134 35439421

[CR133] Singh J, Petter RC, Baillie TA, Whitty A (2011) The resurgence of covalent drugs. Nat Rev Drug Discov 10:307–317 10.1038/nrd3410 21455239

[CR134] Siqueira-Neto JL, Wicht KJ, Chibale K, Burrows JN, Fidock DA, Winzeler EA (2023) Antimalarial drug discovery: progress and approaches. Nat Rev Drug Discov 22:807–826 10.1038/s41573-023-00772-9 PMC10543600 37652975

[CR135] Skinner J, Kayentao K, Ongoiba A, Healy SA, Hu Z, Preston AC, Niangaly A, Schwabl P, Cisse H, Doumbo S et al (2025) Anti-sporozoite monoclonal antibody for malaria prevention: secondary efficacy outcome of a phase 2 randomized trial. Nat Med 31:2682–2690 10.1038/s41591-025-03739-y PMC12353790 40461818

[CR136] Smilkstein MJ, Pou S, Krollenbrock A, Bleyle LA, Dodean RA, Frueh L, Hinrichs DJ, Li Y, Martinson T, Munar MY et al (2019) ELQ-331 as a prototype for extremely durable chemoprotection against malaria. Malar J 18:291 10.1186/s12936-019-2921-9 PMC6712883 31455339

[CR137] Spangenberg T, Burrows JN, Kowalczyk P, McDonald S, Wells TNC, Willis P (2013) The open access malaria box: a drug discovery catalyst for neglected diseases. PLoS ONE 8:e62906 10.1371/journal.pone.0062906 PMC3684613 23798988

[CR138] Stokes BH, Yoo E, Murithi JM, Luth MR, Afanasyev P, da Fonseca PCA, Winzeler EA, Ng CL, Bogyo M, Fidock DA (2019a) Covalent *Plasmodium falciparum*-selective proteasome inhibitors exhibit a low propensity for generating resistance in vitro and synergize with multiple antimalarial agents. PLoS Pathog 15:e1007722 10.1371/journal.ppat.1007722 PMC6553790 31170268

[CR139] Stokes BH, Yoo E, Murithi JM, Luth MR, Afanasyev P, da Fonseca PCA, Winzeler EA, Ng CL, Bogyo M, Fidock DA (2019b) Covalent *Plasmodium falciparum*-selective proteasome inhibitors exhibit a low propensity for generating resistance in vitro and synergize with multiple antimalarial agents. PLoS Pathog 15:e1007722 10.1371/journal.ppat.1007722 PMC6553790 31170268

[CR140] Swearingen KE, Lindner SE (2018) *Plasmodium* parasites viewed through proteomics. Trends Parasitol 34:945–960 10.1016/j.pt.2018.08.003 PMC6204299 30146456

[CR141] Swinney DC (2013) Phenotypic vs. target-based drug discovery for first-in-class medicines. Clin Pharm Ther 93:299–301 10.1038/clpt.2012.236 23511784

[CR142] Takata J, Sondo P, Humphreys GS, Burrow R, Maguire B, Hossain MS, Das D, Commons RJ, Price RN, Guerin PJ (2020) The worldwide antimalarial resistance network clinical trials publication library: a live, open-access database of *Plasmodium* treatment efficacy trials. Am J Trop Med Hyg 103:359–368 10.4269/ajtmh.19-0706 PMC7356478 32431267

[CR143] Tu Z, Choure SJ, Fong MH, Roh J, Levin I, Yu K, Joung JF, Morgan N, Li SC, Sun X et al (2025) ASKCOS: open-source, data-driven synthesis planning. Acc Chem Res 58:1764–1775 10.1021/acs.accounts.5c00155 PMC12140107 40397546

[CR144] Turon G, Hlozek J, Woodland JG, Kumar A, Chibale K, Duran-Frigola M (2023) First fully-automated AI/ML virtual screening cascade implemented at a drug discovery centre in Africa. Nat Commun 14:5736 10.1038/s41467-023-41512-2 PMC10504240 37714843

[CR145] Turon G, Mulubwa M, Montaner A, Njoroge M, Chibale K, Duran-Frigola M (2025) Artificial intelligence coupled to pharmacometrics modelling to tailor malaria and tuberculosis treatment in Africa. Nat Commun 16:9258 10.1038/s41467-025-64304-2 PMC12537934 41115876

[CR146] Turon G, Abel, L, Marina M-L, Arnau CC, Miquel D-F (2024) Ersilia model hub: a repository of AI/ML for neglected tropical diseases. Zenodo

[CR147] van der Pluijm RW, Tripura R, Hoglund RM, Pyae Phyo A, Lek D, ul Islam A, Anvikar AR, Satpathi P, Satpathi S, Behera PK et al (2020) Triple artemisinin-based combination therapies versus artemisinin-based combination therapies for uncomplicated *Plasmodium falciparum* malaria: a multicentre, open-label, randomised clinical trial. Lancet 395:1345–1360 10.1016/S0140-6736(20)30552-3 PMC8204272 32171078

[CR148] Vaughan AM, Pinapati RS, Cheeseman IH, Camargo N, Fishbaugher M, Checkley LA, Nair S, Hutyra CA, Nosten FH, Anderson TJ et al (2015) *Plasmodium falciparum* genetic crosses in a humanized mouse model. Nat Methods 12:631–633 10.1038/nmeth.3432 PMC4547688 26030447

[CR149] Vendrely KM, Kumar S, Li X, Vaughan AM (2020) Humanized mice and the rebirth of malaria genetic crosses. Trends Parasitol 36:850–863 10.1016/j.pt.2020.07.009 PMC7530086 32891493

[CR150] Venture MFM (2025) MMV announces new partnership using AI-powered image analysis to accelerate malaria drug discovery

[CR151] Wall RJ, MacGowan SA, Hallyburton I, Syed AJ, Ajay Castro S, Dey G, Milne R, Patterson S, Phelan J, Wiedemar N et al (2024) ResMAP-a saturation mutagenesis platform enabling parallel profiling of target-specific resistance-conferring mutations in *Plasmodium*. mBio 15:e0170824 10.1128/mbio.01708-24 PMC11481570 39191404

[CR152] Watson JA, Commons RJ, Tarning J, Simpson JA, Llanos Cuentas A, Lacerda MVG, Green JA, Koh G, Chu CS, Nosten FH et al (2022) The clinical pharmacology of tafenoquine in the radical cure of Plasmodium vivax malaria: an individual patient data meta-analysis. Elife 11:e83433 10.7554/eLife.83433 PMC9725750 36472067

[CR153] Webster R, Oyong DA, Llewellyn S, Abd-Rahman AN, Potter AJ, Mukhiya R, Sahai N, Leelasena I, Ujvary E, Mathison S et al (2025) Adjunctive ruxolitinib attenuates inflammation and enhances immunity in volunteers experimentally infected with *Plasmodium falciparum*. Sci Transl Med 17:eaea2531 10.1126/scitranslmed.aea2531 41296825

[CR154] Wellems TE, Panton LJ, Gluzman IY, do Rosario VE, Gwadz RW, Walker-Jonah A, Krogstad DJ (1990) Chloroquine resistance not linked to mdr-like genes in a *Plasmodium falciparum* cross. Nature 345:253–255 10.1038/345253a0 1970614

[CR155] WHO (2025) World malaria report 2025. World Health Organization, Geneva

[CR156] WHO (2023) WHO guidelines for malaria. World Health Organization, Gevena

[CR157] Wicht KJ, Mok S, Fidock DA (2020) Molecular mechanisms of drug resistance in *Plasmodium falciparum* malaria. Annu Rev Microbiol 74:431–454 10.1146/annurev-micro-020518-115546 PMC8130186 32905757

[CR158] Wicht KJ, Woodland JG, Garnie LF, Langeveld H, Taylor D, Godoy LC, Pasaje CFA, de Souza ML, Siqueira-Neto JL, Ghidelli-Disse S et al (2025) The human Chk1 inhibitor CHIR-124 shows multistage activity against *Plasmodium falciparum* via dual inhibition of PfArk1 and hemozoin formation. ACS Chem Biol 21:1738–1748 10.1021/acschembio.6c00264 PMC13386468 42287204

[CR159] Wirth CC, Pradel G (2012) Molecular mechanisms of host cell egress by malaria parasites. Int J Med Microbiol 302:172–178 10.1016/j.ijmm.2012.07.003 22951233

[CR160] Wolff C, Neuenschwander M, Beese CJ, Sitani D, Ramos MC, Srovnalova A, Varela MJ, Polishchuk P, Skopelitou KE, Škuta C et al (2025) Morphological profiling data resource enables prediction of chemical compound properties. iScience 28:112445 10.1016/j.isci.2025.112445 PMC12084007 40384930

[CR161] Woodland JG, Coertzen D, Wicht KJ, Hidalgo VF, Pasaje CFA, Godoy LC, Qahash T, Mmonwa MM, Dziwornu GA, Wambua L et al (2025) The ATM kinase inhibitor azd0156 is a potent inhibitor of *Plasmodium* phosphatidylinositol 4-Kinase (PI4Kbeta) and is an attractive candidate for medicinal chemistry optimization against malaria. Angew Chem Int Ed Engl 64:e202425206 10.1002/anie.202425206 PMC12232896 40317875

[CR162] Wouters OJ, McKee M, Luyten J (2020) Estimated research and development investment needed to bring a new medicine to market, 2009-2018. JAMA 323:844–853 10.1001/jama.2020.1166 PMC7054832 32125404

[CR163] Xie SC, Griffin MDW, Winzeler EA, Ribas de Pouplana L, Tilley L (2023) Targeting aminoacyl tRNA Synthetases for Antimalarial Drug Development. Annu Rev Microbiol 77:111–129 10.1146/annurev-micro-032421-121210 37018842

[CR164] Yoo E, Schulze CJ, Stokes BH, Onguka O, Yeo T, Mok S, Gnadig NF, Zhou Y, Kurita K, Foe IT et al (2020) The antimalarial natural product salinipostin A identifies essential alpha/beta serine hydrolases involved in lipid metabolism in *P. falciparum* parasites. Cell Chem Biol 27:143–157.e145 10.1016/j.chembiol.2020.01.001 PMC8027986 31978322

[CR165] Younis Y, Douelle F, Feng T-S, Cabrera DG, Manach CL, Nchinda AT, Duffy S, White KL, Shackleford DM, Morizzi J et al (2012) 3,5-Diaryl-2-aminopyridines as a novel class of orally active antimalarials demonstrating single dose cure in mice and clinical candidate potential. J Med Chem 55:3479–3487 10.1021/jm3001373 22390538

[CR166] Yu X, Feng G, Zhang Q, Cao J (2021) From metabolite to metabolome: metabolomics applications in *Plasmodium* research. Front Microbiol 11:2020 10.3389/fmicb.2020.626183 PMC7829456 33505389

[CR167] Zack M, Stupichev DN, Moore AJ, Slobodchikov ID, Sokolov DG, Trifonov IF, Gobbs A (2025) Artificial intelligence and multi-omics in pharmacogenomics: a new era of precision medicine. Mayo Clin Proc Digit Health 3:100246 10.1016/j.mcpdig.2025.100246 PMC12381589 40881104

[CR168] Zhang K, Yang X, Wang Y, Yu Y, Huang N, Li G, Li X, Wu JC, Yang S (2025) Artificial intelligence in drug development. Nat Med 31:45–59 10.1038/s41591-024-03434-4 39833407

