# Supplementary material for: New thinking for the next generation of antimalarials
Source: EMBO Mol Med. 2026 Sep 2;18(10):3883–97. doi: 10.1038/s44321-026-00515-4 (PMC13658803; doi:10.1038/s44321-026-00515-4)
Supplement: Supplementary file 1 [file 44321_2026_515_MOESM1_ESM.pdf]

EMBO Molecular Medicine

New Thinking for the Next Generation of Antimalarials

Hannah Asiki, Jason Hlozek, John Woodland, Kathryn Wicht, and Kelly Chibale

Corresponding authors: Kelly Chibale (kelly.chibale@uct.ac.za) , John Woodland (john.woodland@uct.ac.za), Kathryn Wicht (kathryn.wicht@uct.ac.za)

Review Timeline:

Submission Date:	29th May 26
Editorial Decision:	23rd Jul 26
Revision Received:	20th Aug 26
Accepted:	20th Aug 26

Editor: Zeljko Durdevic

Transaction Report:

(Note: With the exception of the correction of typographical or spelling errors that could be a source of ambiguity, letters and reports are not edited. Depending on transfer agreements, referee reports obtained elsewhere may or may not be included in this compilation. Referee reports are anonymous unless the Referee chooses to sign their reports.)

Dear Prof. Chibale,

Thank you for the submission of your manuscript to EMBO Molecular Medicine and please accept my apologies for the unusual delay in getting back to you. We have now received feedback from the two of the three reviewers who agreed to evaluate your manuscript. As the referee #2 will unfortunately not be able to return his/her report in a timely manner, we prefer to make a decision now in order to avoid further delay in the process.

As you will see from the reports below, the referees are overall positive about timeliness and interest of the topic; however, they also raise important criticisms that should be fully addressed in a revised manuscript.

I would also like to ask you to amend the following:
1) Remove the figure form the manuscript file and move its legend to the end of the file.
1) Figures: Upon editorial review we may send the article to our graphic artist who will re-draw the figures for style and clarity. Therefore, please ensure the information shown in the figure is scientifically accurate and upload the file as a PDF (or SVG, or EPS), PowerPoint or Keynote in which the labels and objects are still editable. For figures created using Adobe Illustrator, please send the Illustrator (.ai) file.
2) Please rename the figure to Figure 1 and add callouts in the text.
3) Glossary: The glossary is meant to explain some of the terms used for laymen. Could you please identify terms that may need an "explanation"?
4) Please add "Pending issue". At the end of each article is a box highlighting issues that still need further studies and where research efforts should converge. Could you identify some pending issues?
5) Author contributions: Please specify author contributions in our submission system. CRediT has replaced the traditional author contributions section because it offers a systematic machine-readable author contributions format that allows for more effective research assessment. You are encouraged to use the free text boxes beneath each contributing author's name to add specific details on the author's contribution. More information is available in our guide to authors:
https://www.embopress.org/page/journal/17574684/authorguide#authorshipguidelines
6) Please add relevant sources of funding in our submission system and Acknowledgments.
7) As part of the EMBO Publications transparent editorial process EMBO Molecular Medicine will publish online a Review Process File (RPF) to accompany accepted manuscripts. This file will be published in conjunction with your paper and will include the anonymous referee reports, your point-by-point response and all pertinent

correspondence relating to the manuscript.

You can submit your revised files by logging onto our online manuscript tracking system or simply follow this link:

I hope that the referees' comments do not prove too problematic to address and I look forward to reading your next version.

Yours sincerely,

Zeljko Durdevic

Zeljko Durdevic
Senior Editor
EMBO Molecular Medicine

*** IMPORTANT INFORMATION ***

When submitting your revised manuscript, please include:

1) a .doc formatted version of the manuscript text (including Figure legends and tables)
2) Separate figure files
3) a letter INCLUDING the reviewer's reports and your detailed responses to their comments.

Also, and to save some time should your paper be accepted, please read below for additional information regarding some features of our research articles:

1) Glossary: EMBO Molecular Medicine articles will be accompanied by a glossary explaining some of the terms used for laymen. I identified the following:

____, ____, ____

Could you please help us in identifying terms that may need an "explanation" other terms that we can add to the glossary.

2) For more information: This is a short list of related web links for further consultation by the readers. Could you identify some relevant ones? Examples are patient associations, OMIM related links, databases, authors websites, etc.

3) Pending issues: At the end of each article we will have a box highlighting issues that still need further studies and where research efforts should converge (we call this the Pending issues box). From my reading I would say:

----

but I can see there may be many more. Could you work on this as well?

4) Disclosure and competing interest statement: Please include a statement declaring any competing commercial interests in relation to your submitted work.

5) Please note that we now mandate that all corresponding authors list an ORCID digital identifier. This takes <90 seconds to complete. We encourage all authors to supply an ORCID identifier, which will be linked to their name for unambiguous name identification.

Currently, our records indicate that there is no ORCID associated with your account.

Please click the link below to provide an ORCID:
https://embomolmed.msubmit.net/cgi-bin/main.plex?el=A5EI2BFMv7A2CeEq5Bh1B9ftdT9BeTe5sKue0TP2E1DoASwY
-

Thank you,

Zeljko Durdevic

Zeljko Durdevic
Editor
EMBO Molecular Medicine

***** Reviewer's comments *****

Referee #1 (Remarks for Author):

Asiki and colleagues present an excellent and well informed review of a dynamic area of drug discovery as applied to the world of malaria - the latter representing a huge global need that invariably lags behind technological advances.

My comments are minor and optional suggestions for improving the article for non specialists in this field.

1. The article contains a large number of abbreviations and acronyms to present different methodologies and approaches that are being used and is somewhat hard to digest in totality; an additional figure or summary box to categorise and group some of these utilities would help. Figure 1 provides a welcome overview of the high level concepts and how they relate, but lacked some details within each of the boxes. I would have liked some expansion of boxes to list some of the key examples of new methods that were applied. For instance in "Target-Based screening" - listing IVEWGA, chemoproteomics, QSAR, etc. Likewise in Figure 1 - for the lead optimisation LAIs, TCIs, HDTs might also include Mabs and chimeric molecules, where TACTs may sit within Drug or target repurposing/repositioning. The figure would become too crowded but then an additional box or figure would help.

2. The Key TCPs for malaria are noted on Page3, but conspicuously missing TCP-2. The reference to Siqueria-Neto is useful, but perhaps this important figure (or a version of it) would be useful to have in the malarial context of next generations entities.

3. The article is very much focused on drug discovery, but the Title is "Next generation of antimalarials", hence this extends beyond discovery to optimisation and implementation.

Clinical trials are mentioned but in main perhaps beyond the scope of the review. Perhaps worth noting that nearly all old and also new antimalarial drugs that have been licensed after Phase3, have been recommended at the wrong or suboptimal dose particularly in children where Pk studies are missing. For instance Fansidar (Barnes Clin Pharmacol Ther 2006) and (contentiously) and newly licenced tafenoqoquine (Watson eLife 2022). Figure 1 might include a Clinical Trials Phase I-IV, or post market optimisation. The impact of dose optimisation can be huge 4% better efficacy in African children could prevent 4million cases of malaria per year.

4. Page 11 clinical trials. The reference to AI for clinical trials (Askin 2023) refers to cancer trials and its relevance to malaria trials is less clear. Also although Africa is underrepresented in clinical trials per se it represents the majority of antimalarial trials (Das et al MalariaJ 2013). On Page 7 RCTs are claimed to be difficult to "schedule". I am not sure what that means - but certainly logistically challenging, costly and time consuming - hence emphasising the importance of prioritising suitable candidates in upstream discovery.

Referee #3 (Remarks for Author):

This review by Asiki et al. does a good job at filling an important gap in the landscape of excellent recent reviews on antimalarial drug development by placing an emphasis on potentially valuable/promising new approaches (the current gold standard, Siqueira-Neto et al. 2023, co-authored by two of the authors of this review is looking more into what has worked in the past). It appears quite comprehensive with a few notable exceptions in my opinion. It is well-written and generally well-structured although I would like to suggest adding a few subtitles to avoid a "counting down" of technologies and approaches paragraph by paragraph.

I have only one major comment:

1. Whilst I would not argue with the value of induction of resistance experiments for discovering drug targets, I was missing one important, classical approach - genetic crosses (e.g., discovery of chloroquine resistance and more recent, for discovering genetic modifiers of quinine susceptibility). I accept that these experiments were not conducted as part of a drug discovery pipeline - still I would strongly recommend discussing their value for our understanding of how antimalarials work.

Minor comments:

2. I believe that WHO is very clear about the fact that partial artemisinin resistance is currently a threat but not (yet) an explanation of the remarkable stagnation or even, slight increase of the annual global malaria morbidity and mortality estimates. ACTs still work outside of South-East Asia, which constitutes only a tiny share of the global malaria burden.
3. I would not share the, relatively speaking, pessimistic assessment of the current antimalarial pipeline ("incremental innovation"). There is a lot more going on now than for instance 20-30 years ago (thanks largely to substantially more funding). Maybe a short historical perspective could be useful to better frame this discussion?
4. Liver schizonts do not (by most estimates) release 100,000 merozoites (more typical estimates range from 1,000 to 10,000 per liver stage) - also please add a few more citations for this life-cycle paragraph.
5. Blood stage schizonts do not "rupture", this is a highly coordinated, active process, termed egress.
6. "Concurrently, gametocytogenesis occurs" - please rephrase as this is potentially misleading. Gametocytes do indeed circulate (with I suppose a half-life of around 1 week) but I don't think that "persist" is the right word to describe it.
7. "Historically, most treatments have focused on the asexual blood stage..." - unfortunate wording: (a) usually we treat diseases and (b) only asexual blood stages cause disease - so what else are we supposed to treat?
8. Please explain what TCP-4 is about
9. "Multi-targeted therapies that address both disease treatment and malaria eradication" - see #7 and what exactly is meant by "multi-targeted" therapies?
10. Would be nice to clarify whether the identification of a drug target is mandatory for registration (i don't think so).
11. "SP-amodiaquine" - please spell out SP (unless it had been previously in the text, which I haven't seen)
12. The paragraph starting with "Ultimately, effective malaria medicines..." reads like two paragraphs in one

13. I must say that I wasn't familiar with the expression "chemical matter" and had to look it up (it's also not mentioned on Figure 1) - perhaps it would be good to introduce this terminology?
14. As far as I can tell imatinib has not yet been tested in "clinical trials" (plural), only in a single trial?
15. I'm not convinced about the case for AI in clinical trials of new antimalarial drugs (it seems rather far-fetched). I don't have the impression that our current trial designs are in desperate need of improvement.

Response to Reviewers' Comments:

We thank the reviewers for their helpful and insightful comments on our review manuscript, and are happy to address their comments in full, as detailed below:

Referee #1 (Remarks for Author):

Asiki and colleagues present an excellent and well informed review of a dynamic area of drug discovery as applied to the world of malaria - the latter representing a huge global need that invariably lags behind technological advances.
My comments are minor and optional suggestions for improving the article for non specialists in this field.

1. The article contains a large number of abbreviations and acronyms to present different methodologies and approaches that are being used and is somewhat hard to digest in totality; an additional figure or summary box to categorise and group some of these utilities would help. Figure 1 provides a welcome overview of the high level concepts and how they relate, but lacked some details within each of the boxes. I would have liked some expansion of boxes to list some of the key examples of new methods that were applied. For instance in "Target-Based screening" - listing IVEWGA, chemoproteomics, QSAR, etc. Likewise in Figure 1 - for the lead optimisation LAIs, TCIs, HDTs might also include Mabs and chimeric molecules, where TACTs may sit within Drug or target repurposing/repositioning. The figure would become too crowded but then an additional box or figure would help.
We have included many of the specialist terms in a separate glossary which should aid with general understanding. Additionally, we have expanded on Figure 1 as suggested.

2. The Key TCPs for malaria are noted on Page3, but conspicuously missing TCP-2. The reference to Siqueria-Neto is useful, but perhaps this important figure (or a version of it) would be useful to have in the malarial context of next generations entities.
The nomenclature was updated in the 2017 review by Burrows et.al (https://pmc.ncbi.nlm.nih.gov/articles/PMC5237200/) to retire TCP-2 and update TCP-1 to focus on clearance of ABS without separation of compounds with a rapid effect or long duration of action (old TCP-1 vs TCP-2), hence TCP-2 is not used in our review.

3. The article is very much focused on drug discovery, but the Title is "Next generation of antimalarials", hence this extends beyond discovery to optimisation and implementation.

Clinical trials are mentioned but in main perhaps beyond the scope of the review. Perhaps worth noting that nearly all old and also new antimalarial drugs that have been licensed after Phase3, have been recommended at the wrong or suboptimal dose particularly in children where Pk studies are missing. For instance Fansidar (Barnes Clin Pharmacol Ther 2006) and (contentiously) and newly licenced tafenoqoquine (Watson eLife 2022). Figure 1 might include a Clinical Trials Phase I-IV, or post market optimisation. The impact of dose optimisation can be huge 4% better efficacy in African children could prevent 4million cases of malaria per year.
We thank the reviewer for this important observation. We agree that the concept of a "next-generation antimalarial" should extend beyond discovery of new chemical matter to encompass clinical development, dose optimisation and effective implementation. We have therefore inserted an additional paragraph "Clinical development and dose optimisation" in the section "New frontiers and other promising approaches for next-generation antimalarials" to highlight the importance of clinical pharmacology and PK/PD throughout development and, importantly, after regulatory approval. This is particularly relevant to malaria because the principal target population includes young children, in whom developmental differences in PK can result in substantially different drug exposures from those in adults. As suggested, we now discuss Fansidar (sulfadoxine-pyrimethamine) as an instructive historical example in which young children were subsequently shown to have substantially lower drug exposures at recommended doses (Barnes et al, 2006), and tafenoquine as a more recent example illustrating how post-registration PK/PD analyses can identify opportunities for dose optimisation (Watson et al, 2022). We have also revised Figure 1 to extend the antimalarial development continuum through Phase I–III clinical development, regulatory approval and Phase IV/post-marketing optimisation.

4. Page 11 clinical trials. The reference to AI for clinical trials (Askin 2023) refers to cancer trials and its relevance to malaria trials is less clear. Also although Africa is underrepresented in clinical trials per se it represents the majority of antimalarial trials (Das et al MalariaJ 2013). On Page 7 RCTs are claimed to be difficult to "schedule". I am not sure what that means - but certainly logistically challenging, costly and time consuming - hence emphasising the importance of prioritising suitable candidates in upstream discovery.
Page 11: To the best of our knowledge, the use of AI in clinical trials has been reported in several therapeutic areas (Askin 2023); however, this does not yet include AI usage for antimalarial clinical trials. We have edited the text on page 11 to make this point clearer. We have updated the phrase "where just 3% of global clinical trials occur" to be specific to malaria clinical trials "over 60% of malaria clinical trials occur", and framed the poor understanding of African genetic diversity in this context.

Page 7: We have updated the phrasing from "difficult to schedule" to "logistically challenging, costly, and time consuming, particularly in the resource-limited settings with highest burden of malaria".

Referee #3 (Remarks for Author):

This review by Asiki et al. does a good job at filling an important gap in the landscape of excellent recent reviews on antimalarial drug development by placing an emphasis on potentially valuable/promising new approaches (the current gold standard, Siqueira-Neto et al. 2023, co-authored by two of the authors of this review is looking more into what has worked in the past). It appears quite comprehensive with a few notable exceptions in my opinion. It is well-written and generally well-structured although I would like to suggest adding a few subtitles to avoid a "counting down" of technologies and approaches paragraph by paragraph.
As suggested, we have added some sub-headings to the review.

I have only one major comment:

1. Whilst I would not argue with the value of induction of resistance experiments for discovering drug targets, I was missing one important, classical approach - genetic crosses (e.g., discovery of chloroquine resistance and more recent, for discovering genetic modifiers of quinine susceptibility). I accept that these experiments were not conducted as part of a drug discovery pipeline - still I would strongly recommend discussing their value for our understanding of how antimalarials work.

We thank the reviewer for highlighting this omission. We have added a paragraph on the genetic cross approach so that it now reads as follows:
Page 4 "An important classical approach used to retrospectively determine the target of existing antimalarials is a genetic cross, whereby male and female gametes fuse inside a mosquito's gut, undergoing meiosis and gene mixing. The resulting progeny are then isolated and analysed to map specific genes linked to survival, drug resistance and potential target pathways. However, the execution of genetic crosses in *P. falciparum* is technically challenging and resource-intensive, therefore only four experimental crosses were completed during the first four decades of *P. falciparum* genetics research. However, recent advances using humanised mouse models have enabled several additional crosses, enabling resistance trait mapping for drugs such as piperaquine and quinine.
In the context of current drug development, one of the most effective strategies…"

Minor comments:

2. I believe that WHO is very clear about the fact that partial artemisinin resistance is currently a threat but not (yet) an explanation of the remarkable stagnation or even, slight increase of the annual global malaria morbidity and mortality estimates. ACTs still work outside of South-East Asia, which constitutes only a tiny share of the global malaria burden.
To further clarify, we have updated the phrasing on page 1: "This stagnation reflects a convergence of biological, environmental and systemic pressures, *including climate change and population growth*, as well as the concerning emergence and spread of antimalarial drug resistance." This phrasing further emphasises the range of pressures leading to stagnation and does not lay the blame purely on partial artemisinin resistance but rather a range of factors, of which resistance is one.

3. I would not share the, relatively speaking, pessimistic assessment of the current antimalarial pipeline ("incremental innovation"). There is a lot more going on now than for instance 20-30 years ago (thanks largely to substantially more funding). Maybe a short historical perspective could be useful to better frame this discussion?
We thank the reviewer for this important point and agree that our original wording gave an overly pessimistic impression of the current antimalarial pipeline. Our intention was to emphasise that incremental modification of existing therapies alone will not be sufficient to address emerging resistance and the increasingly demanding product profiles required for malaria elimination, rather than to suggest that the current pipeline itself is predominantly incremental. We have revised this section accordingly and added a short historical perspective in the paragraph beginning: "The contemporary antimalarial pipeline should be viewed against a remarkable transformation in malaria drug discovery over the past two to three decades…"

The revised text highlights the substantial transformation of antimalarial R&D over the past two to three decades, driven by increased investment, the establishment of product-development partnerships such as MMV, large-scale phenotypic screening, and increasingly sophisticated parasite biology and target-validation technologies. We thank the reviewer for prompting us to provide this more balanced historical context.

4. Liver schizonts do not (by most estimates) release 100,000 merozoites (more typical

estimates range from 1,000 to 10,000 per liver stage) – also please add a few more citations for this life-cycle paragraph.

The estimate of merozoites has been adjusted to 20,000 in line with the added citation, and further citations have been included for this paragraph.

5. Blood stage schizonts do not "rupture", this is a highly coordinated, active process, termed egress.

We have updated the wording accordingly: "Merozoites egress from schizonts, perpetuating parasitaemia."

6. "Concurrently, gametocytogenesis occurs" - please rephrase as this is potentially misleading. Gametocytes do indeed circulate (with I suppose a half-life of around 1 week) but I don't think that "persist" is the right word to describe it.

We have rephrased the sentences describing gametocytogenesis.

7. "Historically, most treatments have focused on the asexual blood stage..." - unfortunate wording: (a) usually we treat diseases and (b) only asexual blood stages cause disease - so what else are we supposed to treat?

We have changed the wording to "Historically, most drugs have targeted the asexual blood stage..." This preserves our intended meaning of targeting the ABS as opposed to focus on transmission prevention, mosquito targeting interventions, etc, but without using the word "treatment" to avoid any confusion.

8. Please explain what TCP-4 is about

An explanation has been added.

9. "Multi-targeted therapies that address both disease treatment and malaria eradication" - see #7 and what exactly is meant by "multi-targeted" therapies?

The wording here is to highlight the need for a focus on both treatment and eradication. "Multi-targeted" has been removed for clarity.

10. Would be nice to clarify whether the identification of a drug target is mandatory for registration (i don't think so).

We have added a comment to that effect.

11. "SP-amodiaquine" - please spell out SP (unless it had been previously in the text, which I haven't seen)

SP was spelled out on page 2, but we have spelled it out again here to prevent needing to skip back a few sections to find the meaning.

12. The paragraph starting with "Ultimately, effective malaria medicines..." reads like two paragraphs in one

The first sentences of this paragraph have been moved to the discussion section.

13. I must say that I wasn't familiar with the expression "chemical matter" and had to look it up (it's also not mentioned on Figure 1) - perhaps it would be good to introduce this terminology?

We have included a definition for chemical matter in the glossary.

14. As far as I can tell imatinib has not yet been tested in "clinical trials" (plural), only in a single trial?

Thanks for this observation, we have corrected this error to "a clinical trial" (singular).

15. I'm not convinced about the case for AI in clinical trials of new antimalarial drugs (it seems rather far-fetched). I don't have the impression that our current trial designs are in desperate need of improvement.

While we agree with the reviewer that current antimalarial trials are not in desperate need of improvement, our intention is to highlight examples where AI is already being applied in trials for other disease areas and that could be of use for future antimalarial trials. We have made this clearer in the text on page 11.

20th Aug 2026

Dear Prof. Chibale,

We are pleased to inform you that your manuscript is accepted for publication and is now being sent to our publisher to be included in the next available issue of EMBO Molecular Medicine.

Your manuscript will be processed for publication by EMBO Press. It will be copy edited and you will receive page proofs prior to publication. Please note that you will be contacted by Springer Nature Author Services to complete licensing information.

Should you be planning a Press Release on your article, please notify the production team via this form in order to coordinate publication and release dates: https://forms.cloud.microsoft/e/eH4A8JVP6g

If you have any questions, please do not hesitate to contact the Editorial Office. Thank you for your contribution to EMBO Molecular Medicine.

Yours sincerely,
Zeljko Durdevic

Zeljko Durdevic
Senior Editor
EMBO Molecular Medicine
